# Blood monocyte-derived CD169^+^ macrophages contribute to antitumor immunity against glioblastoma

**DOI:** 10.1038/s41467-022-34001-5

**Published:** 2022-10-20

**Authors:** Hyun-Jin Kim, Jang Hyun Park, Hyeon Cheol Kim, Chae Won Kim, In Kang, Heung Kyu Lee

**Affiliations:** Graduate School of Medical Science and Engineering, Korea Advanced Institute of Science and Technology (KAIST), Daejeon, 34141 Republic of Korea

**Keywords:** CNS cancer, Tumour immunology, Cancer microenvironment, Monocytes and macrophages

## Abstract

Infiltrating tumor-associated macrophages (TAM) are known to impede immunotherapy against glioblastoma (GBM), however, TAMs are heterogeneous, and there are no clear markers to distinguish immunosuppressive and potentially immune-activating populations. Here we identify a subset of CD169^+^ macrophages promoting an anti-tumoral microenvironment in GBM. Using single-cell transcriptome analysis, we find that CD169^+^ macrophages in human and mouse gliomas produce pro-inflammatory chemokines, leading to the accumulation of T cells and NK cells. CD169 expression on macrophages facilitates phagocytosis of apoptotic glioma cells and hence tumor-specific T cell responses. Depletion of CD169^+^ macrophages leads to functionally impaired antitumor lymphocytes and poorer survival of glioma-bearing mice. We show that NK-cell-derived IFN-γ is critical for the accumulation of blood monocyte-derived CD169^+^ macrophages in gliomas. Our work thus identifies a well-distinguished TAM subset promoting antitumor immunity against GBM, and identifies key factors that might shift the balance from immunosuppressive to anti-tumor TAM.

## Abstract (web summary)

Tumor-associated macrophages are believed to promote tumour progression and to hamper immune therapy in gliomas. Here authors identify a distinct population of macrophages within the glioblastoma immune microenvironment with antitumour properties and clearly distinguishable phenotypes and gene expression patterns from tumour promoting macrophages.

## Introduction

GBM is the most aggressive form of brain tumor. One of the biggest hurdles to effective therapy for GBM remains the large accumulation of immunosuppressive macrophages into the tumor, which suppress immune responses and support tumor growth^1^. Macrophages comprise 30% to 50% of the solid tumor mass and support tumor progression through the expression of growth factors, enzymes, and cytokines that mediate tumor growth, angiogenesis, and immunosuppression^2–4^. An abundance of macrophages within the tumor microenvironment (TME) is correlated with a higher tumor grade and a worse prognosis for several tumors^5^. In a recent clinical trial, an immune checkpoint inhibitor (ICI) therapy targeting T cells failed in GBM patients despite its strong efficacy against melanoma^6^. The failure of this and other immunotherapies is largely due to the immunosuppressive TME of gliomas.

During development, tissue-resident macrophages originate from embryonic precursors in the yolk sac and fetal liver. These macrophages are replaced through homeostatic mechanisms by monocyte-derived macrophages in adults, except for some macrophages in brain, liver, and lung^7,8^. Inflammatory signals produced by tumors induce the infiltration of these monocyte-derived macrophages (MDM) into tumor tissues. Tumor-associated macrophages (TAM) are composed of both tissue-resident macrophages and infiltrated MDM^9^. The proportion and function of TAMs with different origins vary by tissue^10^. Microglia, the tissue-resident macrophages of the brain, are maintained through the self-renewal of embryonic precursors and are not replaced by hematopoietic cells during homeostasis. However, MDMs infiltrate into the brain under disease and inflammatory conditions. Microglia and MDM are located in discrete regions within brain tumors and possess distinct gene expression signatures^11^. Reducing MDM infiltration suppresses the growth of various solid tumors and improves survival^12–14^, suggesting that MDM infiltration can promote an immunosuppressive TME. In contrast, depletion or prevention of MDM infiltration into brain tumors reduced the efficacy of ICI therapy in a mouse model^15^. These conflicting results indicate that MDMs are heterogenous populations composed of both tumor-supportive and tumor-suppressive macrophages. Although these subsets of MDMs are attractive targets for immunotherapy, current cellular markers cannot distinguish between these divergent immunomodulatory macrophage populations.

CD169 (Sialoadhesin, Siglec1) is a lectin that recognizes 2,3-sialic acid on cell surface glycoproteins. CD169 contains a long extracellular domain suitable for cell-cell interaction, but CD169 is the only SIGLEC molecule with a short intracellular domain that does not initiate intracellular signaling^16^. This suggests CD169 primarily acts to increase the affinity of macrophages to other cells. CD169 is expressed primarily on macrophages in marginal zone of spleen and subcapsular sinus of LN that capture free-floating antigens in blood or lymph including bacteria, virus, and apoptotic vesicles. Prior studies have shown that CD169^+^ macrophages can mediate T cell responses to pathogens and promote immune cell tolerance to apoptotic cells^17–20^. Resident macrophages in peripheral tissues also express CD169 in homeostasis although monocytes or macrophages exposed to inflammatory signals including interferons (IFN) and lipopolysaccharide (LPS) express CD169 in vitro^21,22^. However, the role and origin of intra-tumoral CD169^+^ macrophages remain unclear.

In this study, we analyze the role of tumor-infiltrating CD169^+^ macrophages from GBM patients and mouse glioma models. Our studies show that brain tumor-infiltrating CD169^+^ macrophages are derived from blood monocytes and can promote antitumor immune responses. We demonstrate that depletion of CD169^+^ macrophages reduce CXCL10 expression and infiltration of activated T cells. In addition to being a marker of anti-tumoral macrophages, CD169 on macrophages mediate the phagocytosis of apoptotic tumor cells. Our data demonstrate that CD169^+^ macrophages promote a proinflammatory TME and support cytotoxic immune cell functions.

## Results

### CD169 is expressed in tumor-infiltrating macrophages from mouse and human gliomas

**Fig. 1:**
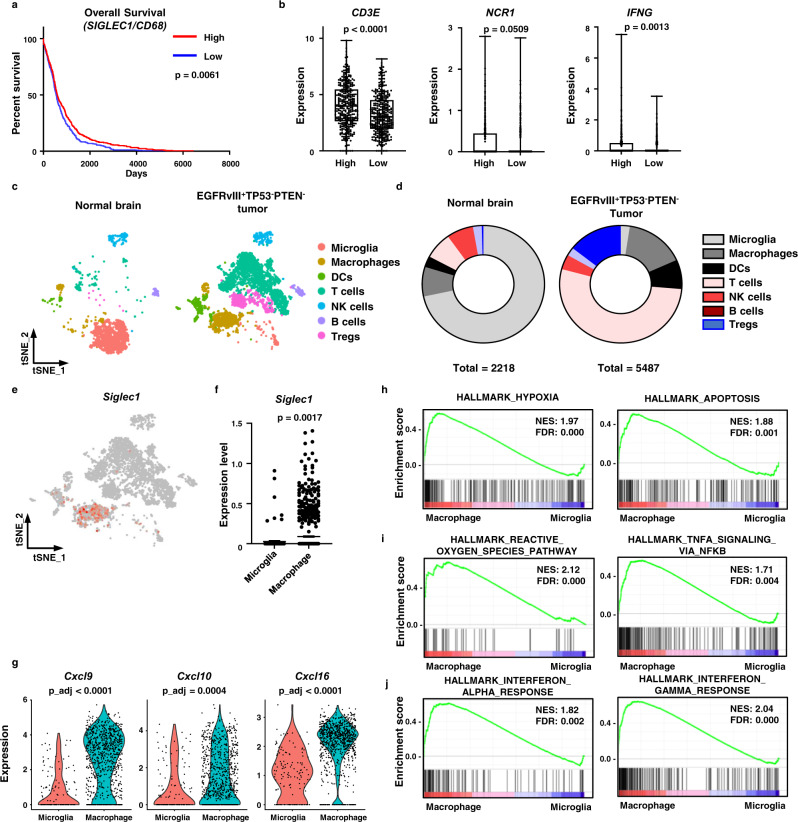
CD169 is a marker of inflammatory macrophages in human and mouse gliomas. **a**, **b** We analyzed human data from The Cancer Genome Atlas (TCGA) Glioblastoma (TCGA-GBM) and Low Grade Glioma (TCGA-LGG) databases (*n* = 694). Patients were grouped by their gene expression levels compared to the median. **a** Overall survival and **b** proinflammatory gene (*CD3E*, *NCR1*, and *IFNG*) expression in patients grouped by high (*n* = 347) or low (*n* = 347) *SIGLEC1* expression in tumor-infiltrating macrophages (*SIGLEC1*/*CD68*, median = 0.632). Difference in survival was measured by log-rank (Mantel-Cox) test. The *p* values for gene expression were calculated by unpaired two-tailed *t*-test. Box plots denote the median, 25–75th percentiles, and minimum and maximum values. **c**–**j** Mouse GBM was induced by deletion of transformation related protein p53 (*Tp53*) and phosphatase and tensin homolog (*Pten*) and by expression of epidermal growth factor receptor variant III (*EgfrvIII*) in the brain. CD45^+^ immune cells were sorted from normal brains and from tumors 16 weeks after gene modification (*n* = 3 in each group) for single-cell RNA sequencing (scRNAseq). **c**, **d** Immune cells were clustered by their gene expression. DCs, dendritic cells; NK cells, natural killer cells; Tregs, regulatory T cells. **e** Expression of *Siglec1* in GBM-infiltrating immune cells. **f** Expression levels of *Siglec1* in microglia and macrophage clusters from tumors. The *p*-value was calculated by unpaired two-tailed *t*-test. Data are presented as mean values + /− SEM. **g** Expression of proinflammatory chemokines (*Cxcl9*, *Cxcl10*, and *Cxcl16*) in tumor-infiltrating macrophages and microglia. p_adj is adjusted p-value for multiple comparisons. Significance was calculated from two-sided Wilcoxon Rank-Sum test. **h**–**j** Gene set enrichment assay (GSEA) with DEGs between tumor-infiltrating macrophages and microglia. NES normalized enrichment score, FDR false discovery rate. Source data are provided as a Source Data file.

To determine if CD169^+^ macrophage infiltration affected patient survival, we analyzed the CD169 expression (*SIGLEC1)* and overall survival of human glioma patients in The Cancer Genome Atlas (TCGA) database. Unexpectedly, the expression of *SIGLEC1* was positively correlated with tumor grade (Supplementary Fig. 1a), and patients with high levels of *SIGLEC1* had lower overall survival (Supplementary Fig. 1b). The expression of *CD68* was also elevated in high-grade gliomas, suggesting increased total numbers of macrophages in the tumor (Supplementary Fig. 1c). Even though the ratio of *SIGLEC1* to *CD68* expression (*SIGLEC1*/*CD68*) correlated with tumor grade (Supplementary Fig. 1d), patients with high *SIGLEC1*/*CD68* ratios had improved overall survival (Fig. 1a). In tumor tissues with a high *SIGLEC1*/*CD68* ratio, there was an increase in the infiltration and function of cytotoxic lymphocytes, based on the expression of the T cell receptor CD3 complex subunit (*CD3E*), natural cytotoxicity receptor (*NCR1*), and interferon gamma (*IFNG*) (Fig. 1b). These results suggested that the increased infiltration of CD169^+^ macrophages promoted an antitumor immune response in human gliomas.

To understand the immunoenvironment of gliomas, tumor-infiltrating immune cells were analyzed by single-cell RNA sequencing (scRNAseq) in two orthotopically injected mouse glioma models (GL261 and CT2A) and in a genetically engineered mouse glioma model (EGFRvIII^+^TP53^−^PTEN^−^, Supplementary Fig. 1e, f). Although microglia are the primary immune cells in the normal brains of C57BL/6 J and FVB mice, tumor progression increased the accumulation of other immune cells into tumors. GBM is known as a ‘cold tumor’ that has restricted T cell responses, but we found T cells largely accumulated into tumors in the late stage of tumor progression (GL261 D20 and EGFRvIII^+^TP53^−^PTEN^−^, Supplementary Fig. 1g). Higher expression levels of immune checkpoint genes in GBM than in normal brain or other glioma samples indicated the exhaustion of tumor-infiltrating T cells (Supplementary Fig. 1h). Macrophages in gliomas were subdivided into six clusters by their gene expression (Supplementary Fig. 1i–j). There were two inflammatory macrophage clusters (2 and 4) and two anti-inflammatory macrophage clusters (1 and 5). In both types of macrophage clusters, each cluster was characterized by different gene expression patterns. Cluster 0 had markers of M2-like TAMs (*C1q* and *Trem2*), cluster 1 had markers of inflammasome activation and antigen presentation (*Nlrp3*, *Il1b*, *Cd74*, and *H2-Aa*), cluster 3 had markers of interferon stimulation in inflammatory macrophages (*Isg*, *Ifit*, and *Cxcl10*), and cluster 5 had functional markers of anti-inflammatory TAMs (*Arg1* and *Ptgs2*).

We analyzed immune cells in genetically engineered mouse gliomas to investigate which macrophages contribute to anti-glioma immunity (Fig. 1c). Infiltrated macrophages expressing *Ly6c2*, a marker of monocyte-derived macrophages, were the major myeloid cells present in the tumor (Fig. 1d and Supplementary Fig. 1f). *SIGLEC1* expression was primarily found in the infiltrated macrophage cluster and was largely absent from the microglial cluster (Fig. 1e, f). Interestingly, tumor-infiltrated macrophages expressed more proinflammatory chemokines and cytokines than microglia within the gliomas (Fig. 1g and Supplementary Fig. 1k, l). Based on gene set enrichment for hypoxia and apoptosis, the TME promoted immunosuppression of the tumor-infiltrated macrophages (Fig. 1h). Furthermore, these macrophages expressed markers of immunosuppressive TAMs (Supplementary Fig. 1m). However, gene sets involved in immune activation, such as the production of reactive oxygen species (ROS) and proinflammatory cytokines, were also more enriched in infiltrated macrophages compared to microglia (Fig. 1i). Enrichment of gene sets for responses to IFN-α and IFN-γ suggested that macrophages were activated by type I and II IFNs (Fig. 1j and Supplementary Fig. 1n). These data implied that macrophages infiltrating into brain tumors are heterogenous cells and are influenced by both immunosuppressive and proinflammatory signals in the TME.

### Tumor-infiltrated CD169^+^ cells are proinflammatory macrophages

**Fig. 2:**
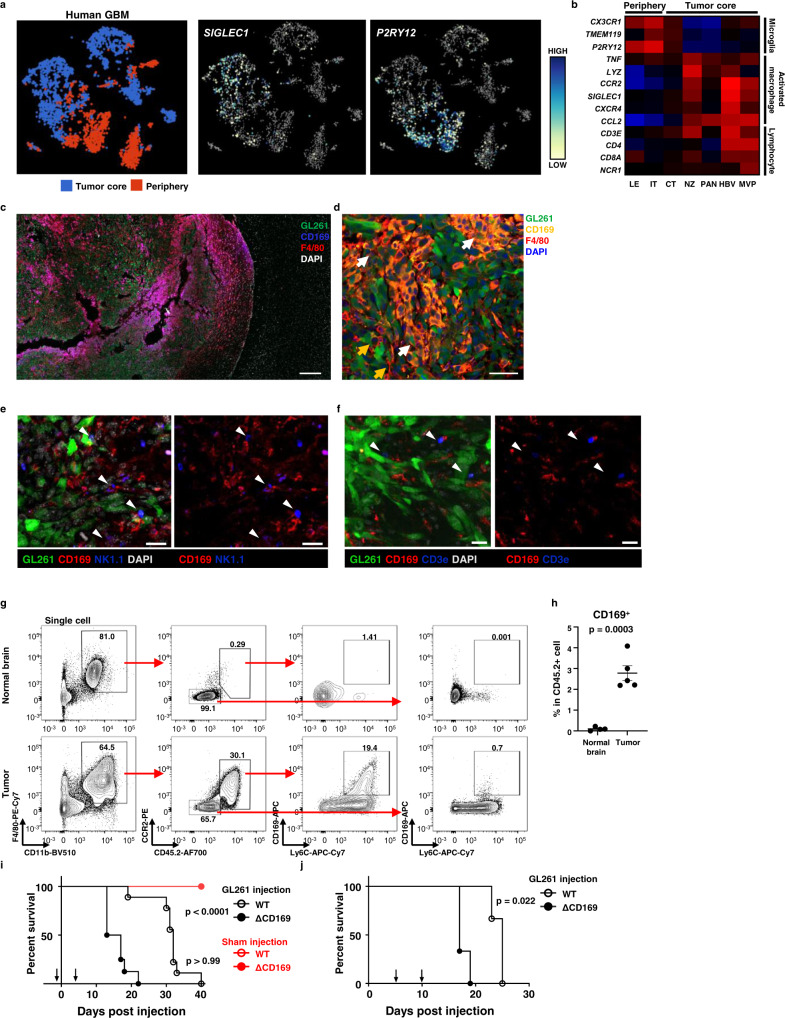
Infiltrated CD169^+^ macrophages promote antitumor immune responses. **a** Analysis of *SIGLEC1* and *P2RY12* in different tumor region of the scRNAseq dataset from human GBM tissues (GSE84465). **b** Gene expression analysis from different regions of GBM (IVY Glioblastoma Atlas Project). RNA sequencing data (FPKM) of 270 tissue blocks from 37 tumors were analyzed for the following markers: microglia (*CX3CR1*, *TMEM119* and *P2RY12*), activated macrophage (*TNF*, *CCL2*, *LYZ*, *CCR2*, *CXCR4* and *SIGLEC1*), and lymphocyte (*CD3E*, *CD4*, *CD8A* and *NCR1*). LE leading edge, IT infiltrating tumor, CT cellular tumor, NZ necrotic zone, PAN pseudopalisading cells around necrosis, MVP microvascular proliferation. **c**, **d** Representative image of a brain tumor (*n* = 3) at 20 days post-injection (dpi) of GL261 glioma cells expressing green fluorescent protein (GFP). **c** The scale bar is 200 μm. **d** White arrows indicate CD169^+^F4/80^+^ cells. Yellow arrows indicate CD169^−^F4/80^+^ cells. The scale bar is 50 μm. Images represent two independent experiments. **e**, **f** Representative images of brain tumor-infiltrating NK cells (**e**) and T cells (**f**) with CD169^+^ macrophages from *n* = 3 biologically independent mouse. Data represent three independent experiments. White arrows indicate co-localized cells. The scale bar is 20 μm. **g**, **h** Flow cytometry analysis for CD169 expression by macrophages in normal brains (*n* = 3) and tumors (*n* = 3) at 20 dpi. **g** Bold numbers in plots indicate the percentage of gated cells. **h** The percentage of CD169^+^ cells in CD45.2^+^ immune cells. The *p*-value was calculated by unpaired two-tailed *t*-test. Data are presented as mean values + /− SEM. Plots and graph represent three independent experiments. **i**, **j** Survival of mice inoculated with intracranial GL261 and intraperitoneal diphtheria toxin (DT; 40 ng per 1 g of mouse body weight). ΔCD169 indicates CD169-depletion group. **i** Wild type (WT) (*n* = 9) and CD169-DTR (*n* = 8) mice were intraperitoneally injected with DT at −1 and +4 days of GL261 injection. DPBS instead of GL261 cells were injected as a sham injection control. **j** WT (*n* = 3) and CD169-DTR (*n* = 3) mice were injected with GL261 and treated with DT at +5 and +10 days after GL261 injection. Differences in survival were analyzed by Log-rank (Mantel-Cox) test. Arrows on *x*-axes indicate the day of DT injection. Data represent more than two independent experiments. Source data are provided as a Source Data file.

Infiltration of CD169^+^ macrophages into brain tumors was confirmed using human scRNAseq data (GEO: GSE84465) to analyze immune cells from different tumor regions (Fig. 2a and Supplementary Fig. 2a)^23^. In the tumor periphery, *PTPRC*^+^*CD68*^+^ immune cells expressed *P2RY12*, a marker of microglia. In the tumor core, immune cells expressed *SIGLEC1* (Fig. 2a). Similarly, RNA sequencing data from human GBM tissues confirmed that microglial genes were expressed in the periphery and that activated macrophage genes and *SIGLEC1* were expressed within the central tumor regions. The tumor core also contained higher numbers of activated T cells and natural killer (NK) cells (Fig. 2b).

To assess the contribution of CD169^+^ macrophages in antitumor responses, we utilized the orthotopic GL261 mouse glioma model. CD169 was expressed in macrophages from the splenic marginal zone, pia mater and perivascular regions of the brain but was not detected in microglia (Supplementary Fig. 2b–d). However, the tumor region contained both CD169^+^ and CD169^−^ macrophages (Fig. 2c, d). NK cells and T cells abutted CD169^+^ macrophages in gliomas, suggesting that tumor-infiltrated macrophages are closely associated with cytotoxic immune cells and are likely involved in antitumor immune responses (Fig. 2e, f), which corresponds to the gene expression patterns seen in human GBM tissues (Fig. 2b). Infiltration of CD169^+^ macrophages into the brain tumor was also confirmed through flow cytometry (Fig. 2g, h). In the normal brain, CD11b^+^F4/80^+^ macrophages were resident CD45^low^CCR2^−^ microglia that did not express CD169. Conversely, the tumor contained both CD45^low^CCR2^−^ microglia and CD45^hi^CCR2^+^ macrophages. A fraction of these tumor-infiltrating Ly6C^+^ macrophages expressed CD169, suggesting that CD169^+^ macrophages were derived from monocytes in the blood.

To determine the role of CD169^+^ macrophages in brain tumors, diphtheria toxin (DT) was administered to mice expressing the diphtheria toxin receptor in CD169^+^ cells (CD169-DTR). DT injection of CD169-DTR mice ablated CD169^+^ cells in lymph nodes (LN) and reduced intratumoral CD169^+^ macrophages but not CD169^−^ macrophages and dendritic cells (DC) (Supplementary Fig. 2e–h). When CD169^+^ macrophages were transiently depleted before GL261 glioma cell inoculation, survival of mice was reduced (Fig. 2i and Supplementary Fig. 2i). Depletion of CD169^+^ macrophages starting at 5 days after GL261 injection also reduced the survival of mice (Fig. 2j and Supplementary Fig. 2j). Reduced survival in mice was confirmed under CD169 depletion conditions in the CT2A glioma model (Supplementary Fig. 2k). These data suggested that CD169^+^ macrophages contribute to antitumor immune responses against glioma.

### CD169^+^ macrophages in glioma are IFN-responsive proinflammatory macrophages

**Fig. 3:**
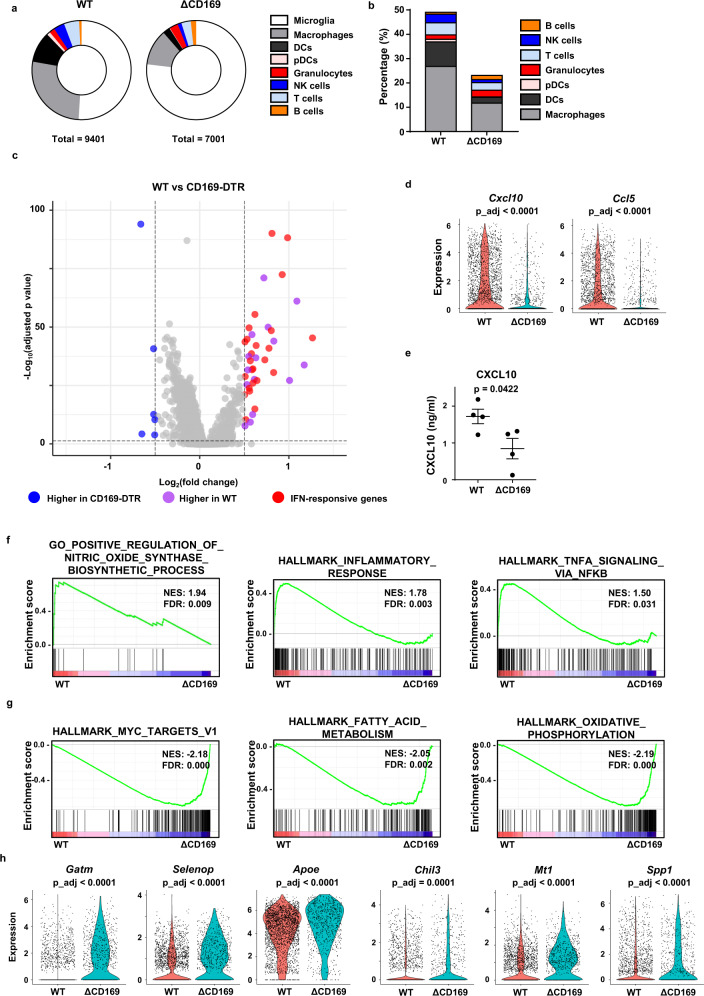
CD169^+^ macrophages are IFN-responsive inflammatory macrophages. Wild-type (*n* = 3) and CD169-DTR (*n* = 3) mice were intracranially injected with GL261. Mice were intraperitoneally injected with DT at −1 and +4 days of tumor injection. Ten days post-inoculation (dpi) of GL261, single cells were isolated and pooled. CD45.2^+^ immune cells were sorted and analyzed by scRNAseq. **a**, **b** Percentage of each immune cell cluster in gliomas of WT and CD169^+^ cell-depleted mice. **c** A DEG plot shows macrophage clusters of WT and CD169-DTR mice (gray dots indicate |log_2_(fold change)| <0.5 or adjusted *p*-value > 0.05). **d** Gene expression of proinflammatory chemokines (*Cxcl10* and *Ccl5*). **e** Expression of CXCL10 in tumor tissues of WT (*n* = 4) and CD169-DTR (*n* = 4) mice. Data represent two independent experiments. The *p* value was calculated from unpaired two-tailed *t*-test. Data are presented as mean values + /− SEM. **f**, **g** Gene sets enriched in infiltrated macrophages from WT and CD169-DTR mice were analyzed. **h** Differentially expressed immunosuppressive tumor-associated macrophage signatures (*Gatm, Selenop, Apoe, Chil3, Mt1*, and Spp*1*) in macrophages from WT and CD169-DTR mice. ΔCD169 indicates CD169-depletion group. All p_adj is adjusted p-value for multiple comparisons, calculated from two-sided Wilcoxon Rank-Sum test. Source data are provided as a Source Data file.

Before we examined the role of CD169^+^ macrophages in glioma, we compared the gene expression of intratumoral CD169^+^ and CD169^−^ macrophages in the GL261 tumor model. As expected, expression of several IFN-stimulated genes was higher in CD169^+^ macrophages (Supplementary Fig. 3a). Analysis of gene set enrichment indicated that CD169^+^ macrophages were responsive to IFN-γ, but these cells did not express type I IFNs (Supplementary Fig. 3b, c). Chemokine CXCL10 expression and enrichment of gene set related to macrophage migration in CD169^+^ macrophages indicated the contribution of CD169^+^ macrophages to immune cell infiltration into glioma (Supplementary Fig. 3a, b). We compared the gene expression of tumor-infiltrating immune cells in the presence and absence of CD169^+^ macrophages to confirm the role of CD169^+^ macrophages in antitumor immune responses. CD169 expression was reduced in the macrophage cluster from CD169-DTR mice, confirming that our DT treatment successfully depleted the majority of CD169^+^ cells (Supplementary Fig. 3d–e). Depletion of CD169^+^ macrophages reduced the overall infiltration of immune cells within the glioma (Fig. 3a, b and Supplementary Fig. 3d). In the analysis of differentially expressed genes (DEG) between macrophage clusters from gliomas in WT and CD169-depleted mice, more than 60% (24/39) were IFN-γ responsive genes (Fig. 3c). Depletion of CD169^+^ macrophages also reduced the expression of *Cxcl10* and *Ccl5*, which are proinflammatory chemokines that recruit T cells and NK cells (Fig. 3d). Reduced CXCL10 expression in gliomas from CD169-DTR mice was also confirmed at the protein level (Fig. 3e). IFN-response genes were also enriched in the macrophage cluster from gliomas in WT mice (Supplementary Fig. 3f). Gene sets for activated macrophages, including nitric oxide synthase (NOS) production, inflammatory response and tumor necrosis factor (TNF-α) signaling, were also enriched in the macrophage cluster from gliomas in WT mice (Fig. 3f). In the macrophage cluster from CD169-depleted gliomas, the enriched gene sets were associated with alternatively activated macrophages and immunosuppressive-macrophage polarization (Fig. 3g, h and Supplementary Fig. 3g)^24^. These data suggest that tumor-infiltrating CD169^+^ cells are macrophages stimulated by IFN-γ that contribute to the proinflammatory tumor microenvironment.

### Depletion of CD169^+^ macrophages reduces T cell responses against gliomas

**Fig. 4:**
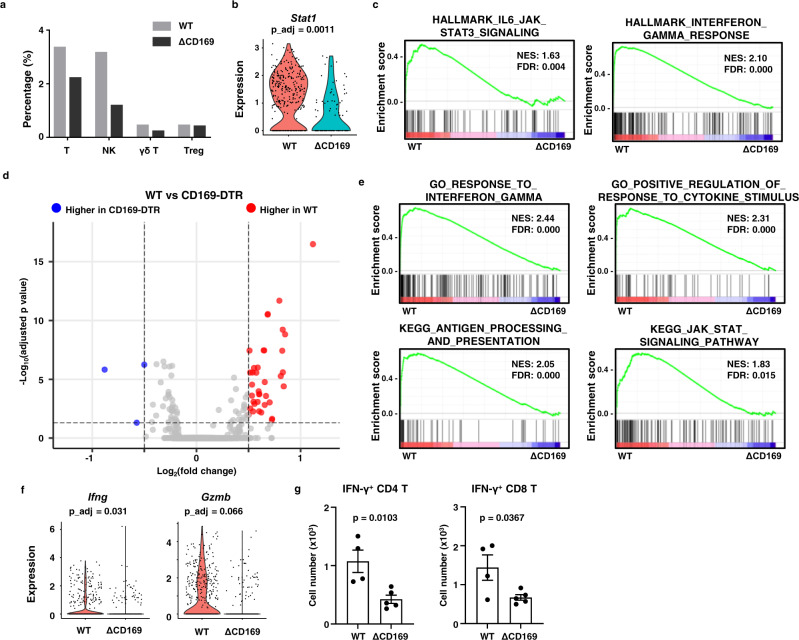
Depletion of CD169^+^ macrophages reduced the tumor infiltration of inflammatory lymphocytes. T cell (*Cd3e*^+^), NK cell (*Ncr1*^+^), γδ T cell (*Trdc*^+^), and Treg (*Foxp3*^+^) were re-clustered in T cell and NK cell clusters (excluding myeloid clusters) and analyzed. **a** Percentage of T cells, NK cells, γδ T cells, and Tregs in *Ptprc*^+^ immune cells were analyzed. **b**, **c** Expression of *Stat1* (**b**) and inflammatory (**c**) gene set enrichment was analyzed for NK cell clusters. **d**–**f** Gene expression of T cell clusters was analyzed using DEG analysis (gray dots indicate |Log_2_(fold change)| <0.5 or adjusted *p*-value > 0.05) (**d**) and gene set enrichment assays (**e**). **f** Expression of *Ifng* and *Gzmb*. **g** IFN-γ-producing glioma-infiltrating T cells were analyzed in WT (*n* = 4) and CD169-DTR (*n* = 5) mice at 10 dpi of GL261 cells. Data represent two independent experiments. ΔCD169 indicates CD169-depletion group. The *p*-values were calculated by unpaired two-tailed *t*-test. Data are presented as mean values + /− SEM. All P_adj for violin plots and volcano plot are adjusted *p* value for multiple comparisons, calculated from two-sided Wilcoxon Rank-Sum test. Source data are provided as a Source Data file.

Properly activated macrophages recruit activated T cells into the tumor through chemokine production^15,25^. In the brain tumors, CD169^+^ macrophages produced proinflammatory chemokines, such as *CXCL10* and *CCL5* (Fig. 3d, e). *Cxcr3* and *Ccr5*, the receptors for CXCL10 and CCL5, were broadly expressed on tumor-infiltrating lymphocytes, including regulatory T cells (Treg), gamma-delta (γδ) T cells, and NK cells (Supplementary Fig. 4a). Depletion of CD169^+^ macrophages reduced the infiltration of T cells and NK cells but did not affect the percentage of γδ T cells or Tregs (Fig. 4a). No significant changes in gene expression were detected in Tregs or γδ T cells (Supplementary Fig. 4b, c). Expression of cytotoxic molecules and receptors were not changed in NK cells following depletion of CD169^+^ macrophages, despite higher expression of genes related to the IFN-γ-response and STAT signaling (Fig. 4b, c and Supplementary Fig. 4d, e). T cells from CD169-depleted mice expressed lower levels of IFN-γ-responsive genes and down-regulated the expression of *Ifng* and *Gzmb* (Fig. 4d–f). Conversely, gene sets associated with T cell activation and antigen-specific responses were enriched in gliomas from WT mice (Fig. 4e). In addition, intratumoral mRNA expression of IL12p40, cytokine critical for T cell activation was significantly decreased under CD169^+^ cell depletion conditions (Supplementary Fig. 4f). These data suggest that the cytotoxic functions of T cells are reduced in the absence of CD169^+^ macrophages.

We confirmed the change in T cell responses after depletion of CD169^+^ macrophages by flow cytometry analysis. IFN-γ producing tumor-infiltrated T cells were reduced in CD169-DTR mice, although the total number of infiltrating CD45^+^ immune cells was comparable with WT mice (Fig. 4g and Supplementary Fig. 4g, h) and IFN-γ producing T cells in the spleen were slightly increased (Supplementary Fig. 4i, j). In line with the scRNAseq analysis, IFN-γ production and expression of CD107a, a marker of degranulation of cytotoxic molecules, in NK cells and γδ T cells was not changed (Supplementary Fig. 4k–m). These results demonstrated that CD169^+^ macrophages promote antitumor immune responses through the accumulation of activated T cells into the tumor.

### CD169^+^ macrophages originated from IFN-γ stimulated blood monocyte-derived macrophages

**Fig. 5:**
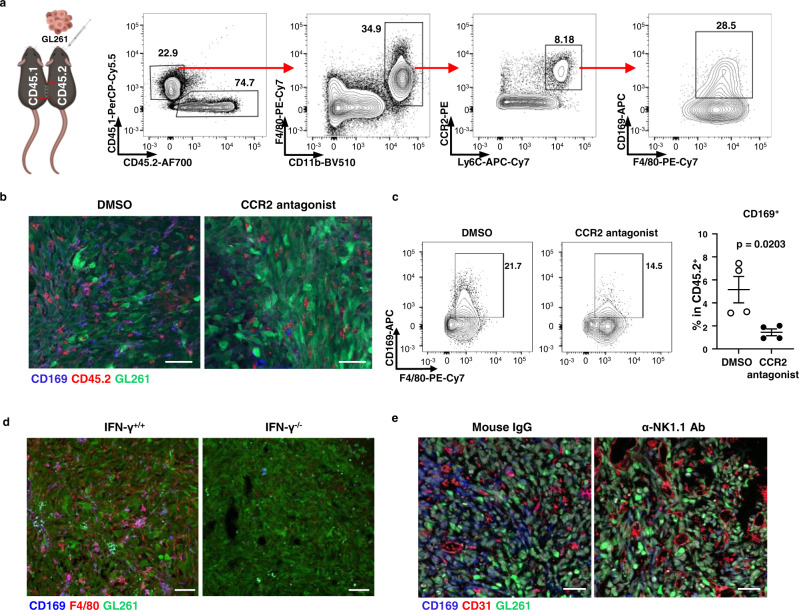
IFN-γ production by NK cells is critical for the tumor infiltration of monocyte-derived inflammatory macrophages. **a** CD45.2 mice (*n* = 3) were connected by parabiosis to CD45.1 mice (*n* = 3) and injected with GL261. Immune cells from the tumor were analyzed at 20 dpi by flow cytometry. The bold numbers in the plots indicate the percentage of gated cells. Data represent two independent experiments. Figure for parabiosis experiment scheme was created with BioRender.com. **b**, **c** C57BL/6 J male mice were treated with 5 mg/kg of CCR2 antagonist every 3 days starting one day before GL261-GFP injection. DMSO was injected as a control. Infiltration of CD169^+^ macrophages into gliomas was analyzed by **b** confocal microscopy at 10 dpi (*n* = 3 for each group) or **c** flow cytometry at 15 dpi (*n* = 4 for each group). **b** Scale bar indicates 50 μm. **c** The *p*-value was calculated by unpaired two-tailed *t*-test. Data are presented as mean values + /− SEM. Data represent three independent experiments. The bold numbers in the plots indicate the percentage of gated cells. **d**, **e** Accumulation of CD169^+^ macrophages in a GL261-GFP derived brain tumor was analyzed by confocal microscopy. Scale bars equal to 50 μm. All image data represent more than two independent experiments. **d** WT (*n* = 3) and IFN-γ KO (*n* = 3) mice were injected with GL261-GFP and analyzed 10 days after tumor injection. **e** C57BL/6 J mice were injected with anti-NK1.1 antibody (*n* = 3) or mouse IgG (*n* = 3) as isotype control at −2, −1, +7 days and analyzed 10 days after tumor inoculation. Scale bars equal to 50 μm. Confocal images represent two independent experiments. Source data are provided as a Source Data file.

In the brain tumor, CD169^+^ macrophages expressed markers of monocyte-derived cells, such as CCR2 and Ly6C (Fig. 2g), although CD169 can be expressed in several tissue-resident macrophages. To confirm the origin of CD169^+^ macrophages in gliomas, we analyzed CD169^+^ macrophages in a parabiosis experiment. A CD45.2 mouse was connected to a CD45.1 congenic mouse and orthotopically implanted with GL261 cells. After glioma formation, CD45.1^+^ cells that had infiltrated from blood into the tumor were analyzed. In the tumor-infiltrating CD45.1^+^ cells, some CD11b^+^F4/80^+^CCR2^+^Ly6C^+^ monocyte-derived macrophages expressed CD169 (Fig. 5a). Blocking monocyte migration with a CCR2 antagonist reduced the infiltration of CD169^+^ macrophages into the tumor (Fig. 5b, c). Diminished infiltration of CD169^+^ macrophages was also confirmed in CCR2 knockout (KO) mice (Supplementary Fig. 5a). These results demonstrated that CD169^+^ macrophages infiltrating the glioma were derived from CCR2^+^ blood monocytes.

Due to the expression of numerous IFN-responsive genes in CD169^+^ macrophages, we hypothesized that tumor-infiltrating macrophages were regulated by IFNs. To confirm this, we analyzed the expression of CD169 in tumor-infiltrating macrophages that were deficient in IFN signaling. CD169 expression was comparable in gliomas from IFNαR1 KO and WT mice (Supplementary Fig. 5b). However, CD169^+^ macrophages were significantly reduced in IFN-γ KO mice (Fig. 5d), suggesting that IFN-γ is critical for the accumulation of CD169^+^ macrophages in gliomas. scRNAseq analysis identified NK cells as the major source of IFN-γ during the early progression of the tumor (Supplementary Fig. 5c). Administration of anti-NK1.1 antibody to deplete NK cells reduced CD169 expression in tumors (Fig. 5e), suggesting that IFN-γ produced by NK cells is required for the infiltration of CD169^+^ macrophages into gliomas.

### CD169 boosts phagocytosis of apoptotic tumor cells and tumor-specific T cell responses

**Fig. 6:**
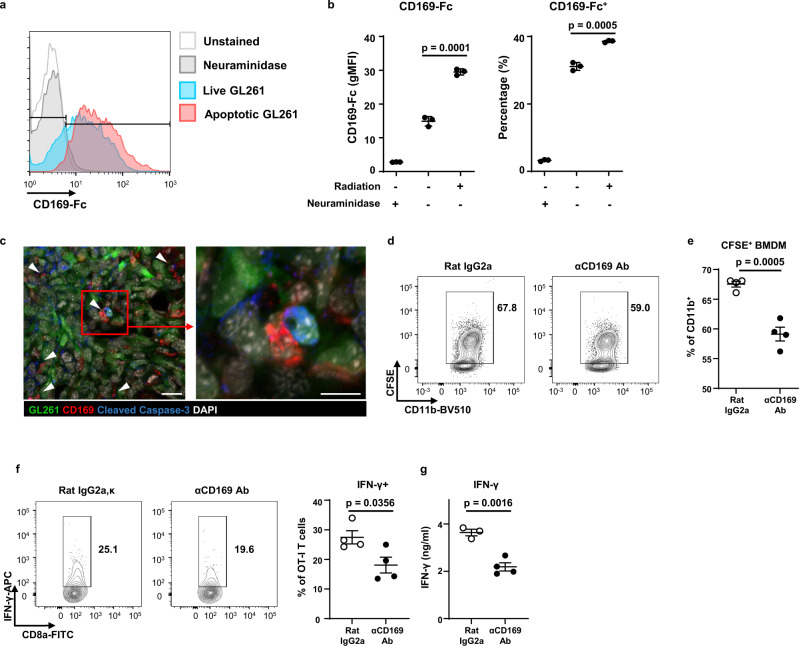
CD169 enhances phagocytosis of apoptotic tumor cells. **a**, **b** Live or apoptotic GL261 were incubated with CD169-Fc and AF647-conjugated anti-mouse IgG to detect CD169 ligand on the cell surface. Neuraminidase was applied before CD169-Fc treatment as a negative control. **a** Cells were analyzed by flow cytometry. **b** Geometric mean fluorescence intensity (gMFI) and percentage of CD169-ligand^+^ cells were measured. Data represent two independent experiments. **c** Co-localization of CD169^+^ macrophages and apoptotic tumor cells expressing cleaved caspase-3 in glioma (*n* = 3 biologically independent mice) was detected by confocal microscopy. The image on the right is a magnified version of the image on the left. Data represent two independent experiments. Scale bars indicate 20 μm for the left image and 10 μm for the right image. White arrows indicate co-localized regions. These images represent two independent experiments. **d**, **e** BMDMs were treated with anti-CD169 blocking antibody or rat IgG as an isotype control before co-culture with apoptotic GL261 tumor cells. **d** Phagocytosis of fluorescently labeled GL261 cells was analyzed by flow cytometry. **e** Percentage of BMDMs that uptake tumor cells. The data represent three independent experiments. **f**, **g** BMDMs were treated with anti-CD169 blocking antibody or rat IgG before phagocytosis of apoptotic GL261 cells expressing ovalbumin (OVA). OT-I T cells were co-cultured with BMDMs for 5 days. **f** IFN-γ-producing T cells were analyzed after phorbol myristate acetate and ionomycin stimulation. **g** IFN-γ produced by OT-I T cells. Bold numbers in plots indicate the percentage of gated cells. The data represent three independent experiments. All *p*-values were calculated by unpaired two-tailed *t*-test. Data are presented as mean values + /− SEM. Source data are provided as a Source Data file.

Previous studies have demonstrated that tumor debris accelerates tumor growth and increases immunosuppression^26–28^. CD169 on macrophages in the spleen and LN mediates the capturing of particles from bacteria, viruses, and dead cells^17,29^. We hypothesized that CD169 contributes to the antitumor immune response by mediating the phagocytosis of tumor cells. Fc-conjugated CD169 (CD169-Fc) bound to GL261 cells, confirming that GL261 glioma cells display the 2,3-sialylated glycoproteins or glycolipids recognized by CD169 (Supplementary Fig. 6a). The binding of CD169-Fc was eliminated by pre-treatment with neuraminidase, which cleaves the glycosidic linkage of sialic acid (Fig. 6a, b). Interestingly, the binding of CD169-Fc to GL261 was increased by the induction of apoptosis in GL261 cells via γ-irradiation. Additionally, CD169^+^ macrophages were co-localized with cleaved caspase-3^+^ apoptotic tumor cells (Fig. 6c). To confirm the contribution of CD169 on the phagocytosis of tumor cells, labeled GL261 cells were co-cultured with CD169-expressing bone marrow-derived macrophages (BMDM) (Supplementary Fig. 6b). The phagocytosis of apoptotic GL261 cells was significantly decreased by blockade of CD169 on BMDMs (Fig. 6d, e). We confirmed that CD169-blockade did not affect the BMDM phagocytosis of latex beads, which lack the CD169 ligand (Supplementary Fig. 6c). These results revealed that tumor-infiltrating macrophages promote an antitumor response through CD169 binding and phagocytosis of dead glioma cells.

Recognition and phagocytosis of dead tumor cells by antigen-presenting cells is the first step to trigger tumor-specific T cell responses^30^. CD169^+^ macrophages directly contacted intratumoral T cells, and depletion of CD169^+^ macrophages reduced the IFN-γ-producing T cells (Fig. 2f). Thus, we hypothesized that phagocytosis enhanced by CD169 would improve antigen-specific T cell responses. To confirm this, we co-cultured BMDMs under CD169 blockade with OT-I T cells; the BMDMs engulfed apoptotic GL261 cells that expressed ovalbumin (GL261-OVA) (Supplementary Fig. 6d). As expected, CD169 blockade during phagocytosis reduced the T cell stimulation by macrophages and the IFN-γ production by antigen-specific T cells (Fig. 6f–g). Taken together, we show that CD169 on macrophages contributes to phagocytosis through ligand binding on apoptotic tumor cells and enhances tumor-specific T cell responses.

## Discussion

GBM, the most aggressive form of brain tumors, is categorized as a ‘cold tumor’ due to the limited T cell responses and a large accumulation of immunosuppressive TAMs^6,31^. We demonstrated that in human GBMs, increased CD169 expression in CD68^+^ macrophages correlated with increased numbers of cytotoxic lymphocytes and increased patient survival. In a mouse glioma model, brain tumor-infiltrating macrophages also expressed CD169 and produced the proinflammatory chemokines CXCL9, CXCL10, and CXCL16. Previous studies have shown that tumor infiltration of activated T cells and NK cells is significantly reduced in mice deficient in CXCR3, the receptor for CXCL9 and CXCL10^32^. Similarly, depletion of CD169^+^ macrophages in our studies reduced the expression of CXCL10 and the infiltration of T cells and NK cells. Furthermore, when we depleted CD169^+^ macrophages, the remaining macrophages displayed an immunosuppressive M2 phenotype. Our results demonstrate that CD169^+^ macrophages in gliomas are proinflammatory and can mediate the accumulation and antitumor responses of T cells and NK cells. CXCR3 signaling in the TME was previously shown to be critical for the responsiveness of immunotherapy^33^, suggesting that CD169^+^ macrophages could be an attractive target for improving the success of immunotherapy treatments against gliomas.

CD169^+^ macrophage depletion reduced the infiltration of a variety of immune cells into tumors. Although few intratumoral macrophages expressed CD169, CD169^+^ macrophage depletion reduced total macrophage infiltration by half. As macrophages produce various chemokines, the depletion of CD169^+^ macrophages reduced intratumoral chemokines and influx of immune cells. It has been reported that intratumoral IFN-γ is important for tumor infiltration of macrophages or DCs and suppresses inhibitory immune cells, thus restricting immune-cell influx into tumors^34,35^. CD169^+^ macrophage depletion decreased IFN-γ-producing T cells in tumors, which may change the immune environment in tumors.

Microglia, resident macrophages in brain parenchyma, and border-associated macrophages (BAM), resident macrophages in brain meninges and perivascular regions, originate from yolk sac precursors and are maintained through self-renewal in homeostasis^36^. CD169 is a marker of several tissue-resident macrophages in non-lymphoid organs as well as lymphoid organs^21,22,37^. Interestingly, microglia did not express CD169 although BAMs expressed CD169 without any inflammatory signals. In this study, we found that CD169^+^ macrophages largely infiltrated into brain tumor. These cells originated from CCR2-dependent blood monocytes and the expression of CD169 was IFN-γ dependent. In contrast to inflammatory phenotype of CD169^+^ macrophages in glioma, perivascular macrophages suppress inflammation by restraining prostaglandin E_2_ production from endothelial cells in LPS stimulation^38^.

IFNs are important cytokines for the induction of CD169 expression^39^. CD169^+^ macrophages in the subcapsular sinuses of LNs are co-localized with IFN-α producing macrophages and plasmacytoid DCs. IFN-γ can also induce CD169 expression in blood monocytes and macrophages in vitro. In this study, the expression of CD169 in gliomas did not change in the absence of IFN-α but was abolished in IFN-γ KO mice. Innate immune cells are the major producer of IFN-γ prior to the activation of adaptive immune responses. We demonstrated that depletion of NK cells, which are major producers of IFN-γ during the early onset of brain tumor progression, reduced the infiltration of CD169^+^ macrophages. Recent studies also suggested that γδ T cells can produce IFN-γ early in the tumor progression before the TME becomes hypoxic^40^. These data suggest that IFN-α and IFN-γ differentially control the expression of CD169 on macrophages under homeostatic and disease conditions.

Because dead cell-derived molecules, such as prostaglandin E (PGE) and adenosine, are known to promote an immunosuppressive TME^27^, clearance of dead tumor cells may indirectly promote cytotoxicity of T cells by creating a more proinflammatory TME. Furthermore, we show that macrophages that engulfed more apoptotic GL261-OVA cells activated more antigen-specific T cells. We also show that blockade of CD169 reduced the levels of phagocytosis, suggesting that phagocytosis is triggered by scavenger receptors, and CD169 probably increases affinity for dead tumor cells. Although cross-presentation by macrophages has also been reported, macrophages are known to be less specialized in cross-presentation than DCs, and the pathway by which phagocytosed antigens are cross-presented has not been well described^41,42^. It will be helpful to investigate the relationship between CD169-enhanced phagocytosis and cross-presentation to clarify the mechanism of cross-presentation by macrophages.

This study identified CD169 as a specific marker for a proinflammatory macrophage subset that enhances the recruitment of cytotoxic immune cells in gliomas. IFN-γ produced from NK cells was shown to be important for the tumor infiltration of CD169^+^ macrophages, suggesting that targeting this pathway could increase the proportion of CD169^+^ macrophages and, thereby, promote a proinflammatory TME within gliomas. Future studies will explore therapies that specifically target tumor infiltration and CD169^+^ macrophage activation.

## Methods

### Mice

Eight- to twelve-week-old age- and sex-matched mice of C57BL/6 J background were used for these experiments unless otherwise specified. C57BL/6 J mice were purchased from KAIST and DBL Co. Ltd (Eumseong, Korea). CD169-DTR (Siglec1^tm1(HBEGF)Mtka^) mice were originally generated and kindly provided by Dr. Masato Tanaka (Tokyo University of Pharmacy and Life Sciences, Japan)^43^. IFN-γ^−/−^ (B6.129S7-Ifng^tm1Ts^/J, Stock No: 002287), IFN-αR^−/−^(B6(Cg)-Ifnar1^tm1.2Ees^/J, Stock No: 028288), CX_3_CR-1^GFP^ (B6.129P2(Cg)-Cx3cr1^tm1Litt^/J, Stock No: 005582), CD45.1 (B6.SJL-Ptprc^a^ Pepc^b^/BoyJ, Stock No: 002014), CCR2^−/−^ (B6.129S4-Ccr2^tm1Ifc^/J, Stock No: 004999), and OT-I (C57BL/6-Tg[TcraTcrb]1100Mjb/J, Stock No:003831) mice were purchased from The Jackson Laboratory (Harbor, ME, USA). LSL-EGFRvIII mice (FVB strain background) were kindly provided by Dr. Jeong Ho Lee (KAIST, Korea). Mice used in this study were maintained in a specific pathogen-free facility of KAIST Laboratory Animal Resource Center. Mice were housed in a specific pathogen-free (SPF) facility at KAIST, in a 12 h/12 h light/dark cycle at 18–24 degrees Celsius and 30–70% of humidity range. Control mice were separately housed in same facility. All procedures in this study were performed in accordance with guidelines and protocols (KA2017-41, KA2019-65, and KA2021-048) approved by the Institutional Animal Care and Use Committee (IACUC) of KAIST. Mice were euthanized with CO_2_ supply when they lost more than 30% of bodyweight or showing abnormal behavior as approved by IACUC of KAIST.

### Cell lines

The GL261 mouse glioma cell line and GFP-expressing GL261 (GL261-GFP) were provided by Dr. Injune Kim (KAIST). We purchased the CT2A mouse glioma cell line (Sigma–Aldrich, St. Louis, MO, USA) and the Lenti-X™ 293 T cell line (Takara Bio, Kusatsu, Japan). L929 cell line producing M-CSF was provided by A. Iwasaki (Yale University, New Haven, CT, USA). Cells were passaged using trypsin-ethylenediaminetetraacetic acid (EDTA) (Corning, Corning, NY, USA) and maintained in Dulbecco’s Modified Eagle Medium (DMEM) complete media (CM, containing 10% of fetal bovine serum (FBS) and 1% penicillin-streptomycin (Corning)) at 37 °C. No mycoplasma contamination was confirmed with e-Myco plus Mycoplasma PCR kit (Intron Biotechnology, Seongnam, Korea). GL261 cells expressing intracellular ovalbumin (GL261-OVA) were maintained in DMEM CM with 1 μg/ml puromycin (Sigma–Aldrich).

### Construction of OVA-containing lentivirus and lentiviral transduction of GL261

OVA DNA (pcDNA3-OVA; Addgene, Watertown, MA, USA) was amplified with the following primers: 5′-AAT TGG ATC CTT AAG CTT GCC ACC ATG G-3′ and 5′-TTA TGA ATT CCT CGA GTT AAG GGG AAA CAC ATC TGC C-3′. OVA-encoding DNA fragments were digested with BamHI and EcoRI (Enzynomics, Daejeon, Korea) and inserted into the third-generation lentiviral plasmid pLV-EF1a-IRES-Puro (Addgene).

For assemble of lentivirus, Lenti-X™ 293 T cells were transfected with the OVA-encoding pLV plasmid and packaging plasmids (pRSV-Rev, pMDLg/pRRE, and pMD2.G; Addgene) using the Lipofectamine 3000 transfection reagent (Thermo Fisher Scientific, Hampton, NH, USA). The culture supernatant of transfected cells was filtered with 0.45 μm cellulose acetate filters (Sartorius, Göttingen, Germany).

GL261 tumor cells were spinoculated with OVA-encoding lentivirus and protamine sulfate (Sigma–Aldrich) by centrifugation at 1000 × *g* and 32 °C for 1 h. Transduced cells were selected with puromycin. To make single clones of GL261-OVA, transduced cells were diluted, plated at 0.5 cells/well, and incubated. Cells from wells containing a single colony were harvested, and OVA expression was confirmed with FITC-conjugated anti-OVA antibody (Abcam, Cambridge, United Kingdom) via flow cytometry.

### Mouse GBM model

Genetically modified mouse gliomas were induced as described^40^. Plasmid DNA encoding small guide RNA for *TP53* and *PTEN*, and the genes for Cas9 and Cre-recombinase, provided from Dr. Jeong Ho Lee (KAIST, Korea), were amplified in DH5α (Enzynomics) and purified with NucleoBond Xtra Midi kit (Takara Bio) following the manufacturer’s instructions. One to two-day-old pups of LSL-EGFRvIII mice were anesthetized by hypothermia. The right lateral ventricle was injected at a depth of 2 mm from the skull with 1 μl of plasmid solution (2 μg/μl). To visualize the DNA injection into the ventricle, Fast Green (Sigma–Aldrich) was added to a final concentration of 1 mg/ml. Mice were subjected to electrical pulses (100 V, 50 ms with 950 ms interval, 5 times) with a NEPA21 Super Electroporator (NEPAGENE, Chiba, Japan). Pups were kept warm during recovery and then returned to their mother. Mice were euthanized 16 weeks after inoculation of DNA for immune cell analyses.

For the orthotopic glioma injection model, 8 to 12-week-old male mice were injected with 1 × 10^5^ GL261 or CT2A cells as described^40^. Tumor cells were detached with a trypsin-EDTA solution and washed twice with Dulbecco’s phosphate buffered saline (DPBS) to remove the residual medium. Cells were resuspended in DPBS (10^5^ cell/ 2 μl). Mice were anesthetized with vaporized isoflurane and fixed on a stereotaxic instrument (Stoelting Co, Wood Dale, IL, USA). The eyes were covered with ointment for protection. The scalp was sterilized with alcohol wipes, and a midline incision was made from the ears to eyes to detach the scalp from the skull. A burr hole was drilled on the skull, 2 mm lateral and 2 mm posterior from bregma. Tumor cells were injected into the right frontal cortex, 3 mm beneath the brain surface, at a rate of 0.4 μl/min for 5 min with a 10 μl Hamilton syringe (The Hamilton Company, Reno, NV, USA) loaded on Nano-injector (KD Scientific, Holliston, MA, USA). DPBS (2 μl) without tumor cells was injected as a sham injection control. After injection, the incision was closed with 7 mm wound clip (Roboz, Gaithersburg, MD, USA), and mice recovered on a heating pad with supplemental oxygen.

### Mice treatment

CD169-DTR heterozygous or wild-type (WT) male mice were intraperitoneally injected with 40 ng/g of diphtheria toxin (DT) (List Labs, Campbell, CA, USA) diluted in 200 μl DPBS at early stages (a day before and 4 days after tumor injection) and intermediate stages (5 and 10 days after tumor injection) of tumor progression.

For NK cell depletion, C57BL/6 J male mice were intraperitoneally injected with 500 μg of anti-mouse NK1.1 antibody (Bioxcell, Lebanon, NH, USA) diluted in 200 μl DPBS. Mouse IgG2a (Bioxcell) was used as an isotype control. Injections were performed 1 and 2 days before and 7 days after tumor injection.

To interfere with monocyte migration, C57BL/6 J male mice were intraperitoneally injected with 5 mg/kg of CCR2 inhibitor RS102895 (Sigma–Aldrich) diluted in 200 μl DPBS starting one day before tumor injection and repeated every 3 days.

### Parabiosis experiment

C57BL/6 J (CD45.2) and congenic CD45.1 female mice were conjoined as described^44^. Female 6-wk-old mice were anesthetized by inhalation of vaporized isoflurane for all procedures. Hair from the right or left side of the body was thoroughly removed for each mouse. Shaved areas were aseptically prepared using betadine and alcohol wipes. Mice were injected with 10 mg/kg Ketoprofen (Ubtech, Anyang, Korea) diluted in 200 μl DPBS for analgesia. Skin from 5 mm below the knee joint to 5 mm above the elbow was incised and detached from the subcutaneous fascia. After tying the elbow and knee joints of each mouse pair, skins of the mice were connected by Surgifit 5-0 sutures (Ailee Co., Seoul, Korea). DPBS (0.5 mL) was subcutaneously injected to prevent dehydration after surgery. The whole incision site was covered with betadine for sanitization. Mice were placed on a heating pad with O_2_ supply until fully recovered. Mice were supplied with drinking water containing 2 mg/ml sulfamethoxazole and 0.4 mg/ml trimethoprim (Tokyo Chemical Industry, Tokyo, Japan) for 10 days and intraperitoneally injected with 10 mg/kg Ketoprofen every day for 3 days to prevent infection and pain. Betadine was applied to the incision every day for 3 days. Experiments were performed 4 weeks after parabiosis.

### Bone marrow-derived macrophages preparation

Bone marrow (BM) was isolated by flushing the femurs and tibiae of 8-week-old C57BL/6 J male mice and passed through a 70 μm strainer. Red blood cells were removed by incubation in Ammonium-Chloride-Potassium (ACK) lysis buffer at room temperature for 5 min. Cells were incubated at 37 °C for 7 days in RPMI 1640 (Corning) CM supplemented with M-CSF containing media (supernatants from a cultured L929 cell line expressing M-CSF).

### Analysis of data from human GBM patient

The TCGA-GBM and TCGA-LGG study data was processed using the Xena platform of UCSC^45^. Gene expression and overall survival data of 694 patients were analyzed. Patients were grouped by high (*n* = 347) or low (*n* = 347) expression of *SIGLEC1* or ratio of *SIGLEC1* and *CD68* (*SIGLEC1/CD68*), compared to median (*SIGLEC1* = 6.82, *SIGLEC1/CD68* = 0.632).

For gene expression analyses of anatomic regions from human GBM, data were gained from IVY Glioblastoma Atlas Project^46^. RNA sequencing data (FPKM) of 270 tissue blocks from 37 tumors were analyzed.

Human GBM scRNAseq data set, GSE84465, was analyzed using BioTuring Bbrowser2 software, version 2.8.22 for Window OS (BioTuring Inf., San Diego, CA, USA)^23,47^.

### Single cell preparation

Mice were transcardially perfused with 50 ml cold DPBS to remove the blood before isolating tissues. The right hemisphere of the brain was minced into small pieces by blade and digested with 2 mg/ml collagenase IV (Worthington Biochemical Corporation, Columbus, OH, USA) and 30 μg/ml DNase I (Roche, Basel, Switzerland) at 37 °C for 30 min. Tissues were passed through a 70 μm strainer. To remove non-immune cells from tumor samples, cells were resuspended in 4 ml of 30% Percoll (GE Healthcare, Chicago, IL) containing DMEM CM and loaded onto 3 ml of 70% Percoll containing DPBS supplemented with 1% bovine serum (Thermo Fisher Scientific). Immune cells and myelin were separated by centrifugation. Cells were treated with ACK lysis buffer for 5 min at RT to remove residual red blood cells.

### Flow cytometry

Single cells were resuspended in staining buffer (DPBS with 1% bovine serum and 1% penicillin-streptomycin) with anti-CD16/32 (clone 2.4G2, ATCC HB-197) (1:100 diluted) to block Fc receptors and stained with antibody mixture.

For CD169 macrophages, 1:200 diluted CD169 (Siglec1)-APC (3D6.112) (Biolegend, San Diego, CA, USA), and 1:300 diluted CD45.2-Alexa Fluor 700 (104), Ly6C-APC-Cy7 (HK1.4), CD192 (CCR2)-PE (SA203G11), F4/80-PE-Cy7 (BM8) (Biolegend) and CD11b-BV510 (M1/70) (BD Biosciences, Bergen County, NJ, USA) were added. CD45.1-PerCP-Cy5.5 (A20) (BD Biosciences) (1:300 diluted) was added for parabiosis experiment. 1:1000 diluted propidium iodide (PI) (Biolegend) or 1:400 diluted BD Horizon Fixable Viability Stain 450 (FV450) (BD Biosciences) was added to exclude dead cells.

For DCs, we used the 1:300 diluted CD45.2-Alexa Fluor 700 (104), CD11c-PE-Cy7 (N418) (Biolegend), F4/80-PerCP-Cyanine5.5 (BM8) (Thermo Fisher Scientific), CD11b-BV510 (M1/70) and 1:1500 diluted MHC II-BV421 (M5/114) (BD Biosciences). PI (1:1000 diluted) was added before acquisition to exclude dead cells.

For T lymphocytes, 1:300 diluted CD45.2-Alexa Fluor 700 (104) (Biolegend), CD8a-FITC (53-6.7), CD4-PerCP-Cy5.5 (GK1.5), CD3e-PE-Cy7 (145-2C11) and CD11b-BV510 (M1/70) (BD Biosciences) were used. FV 450 was added to exclude dead cells.

For NK cells and γδ T cells, 1:300 diluted γδ TCR-FITC (GL3), F4/80-PerCP-Cy5.5 (BM8) (ThermoFisher Scientific), NK1.1-PE (PK136), CD45.2-Alexa Fluor 700 (104) (Biolegend) were added. FV450 was added to exclude dead cells.

For intracellular IFN-γ staining, isolated immune cells were stimulated with 50 ng/ml Phorbol 12-myristate 13-acetate (Sigma–Aldrich), 1 µg/ml ionomycin (Sigma–Aldrich), and 2 µM GolgiStop (BD Biosciences) for 3 h. For analysis of degranulation, 1:100 diluted CD107a-PE-Cy7 (1D4B) or rat IgG2a-PE-Cy7 isotype control (RTK2758) (Biolegend) was added before stimulation. After surface staining, cells were fixed and permeabilized with the Fixation/Permeabilization Solution Kit (BD Biosciences) according to the manufacturer’s instructions and stained with 1:100 diluted IFN-γ-PE (for T lymphocytes) or IFN-γ-APC (for NK cells and γδ T cells) (XMG1.2) (Biolegend). Rat IgG1a-PE or Rat-IgG1a-APC were used as isotype control. Cells were washed with staining buffer and analyzed by flow cytometry on LSR Fortessa (BD Biosciences) using FACSDiva software (BD Biosciences, version 8.0.2) or FACS Calibur (BD Biosciences) using CellQuest Pro software (BD Biosciences, version 6.0). Data were analyzed by FlowJo version 10 software (BD Biosciences).

### Single cell RNA sequencing analysis

Single cells isolated from normal brain or tumor tissues were stained with 1:300 diluted CD45.2-APC (104) (BD Biosciences) and 1:1000 diluted PI. CD45.2^+^PI^−^ live immune cells were sorted on a FACS ARIA II cell sorter (BD Biosciences). The 10x chromium single cell 3’ library kit (10x Genomics, Pleasanton, CA, USA) was used to generate a single cell library. Samples were sequenced with HiSeqXten (Illumina, San Diego, CA, USA) for 10,000 cells. The count matrix was generated using Cell Ranger (10x Genomics). Downstream analysis was performed using Seurat v3^48^. For data analysis of the GL261 gliomas, cells with an RNA count too low (<200) or mitochondrial gene expression too high (>17%) were excluded. For data of genetically engineered gliomas, cells with RNA feature counts too low (<1500) or mitochondrial gene expression too high (>10%) were excluded. For CT2A glioma, cells with an RNA count too low (<200) or mitochondrial gene expression too high (>10%) were excluded. Data from each set were integrated using FindIntegrationAnchors (dims = 1:50) and IntegrateData (dims = 1:50) functions. Data were normalized using the LogNormalize method with a 10,000 scale factor. The gene expression is given as the natural log of (1+normalized read count). Clusters were separated using FindVariableFeatures (nfeatures = 2000), FindNeighbors (dims = 1:30), and FindClusters functions (resolution = 1); 17,647 cells from GL261 tumors and 7705 cells from genetically engineered gliomas were estimated following these processes. For a detailed analysis of cytotoxic lymphocytes in GL261 gliomas, clusters of T cell and NK cell were isolated and re-clustered using FindNeighbors (dims = 1:20) and FindClusters functions (resolution = 0.5). For analysis of macrophages in gliomas, we isolated and re-clustered macrophage clusters from GL261, CT2A, and genetically engineered glioma samples using the FindNeighbors (dims = 1:10) and FindClusters functions (resolution = 0.5). Clusters expressing *Cd3e* and *P2ry12* were excluded to remove contamination of lymphocytes and microglia. DEGs among clusters and samples were analyzed using the FindAllMarkers function and visualized using the EnhancedVolcano ver.1.13.2 package^49^. Gene set enrichment and gene ontology were analyzed with GSEA software, a joint project of UC San Diego and Broad Institute^50,51^.

### CXCL10 measurement in tumor tissue

The right cerebrum was isolated 10 days after tumor injection and homogenized by passing through a 70 μm strainer (SPL, Pocheon, Korea) with 1 ml DPBS. Cells and myelin were removed by centrifugation and supernatants were collected (pellets were used for RNA extraction). CXCL10 levels in the supernatant were measured using CXCL10-DuoSet (R&D Systems, Minneapolis, MN, USA) following the manufacturer’s instructions. Brain tumor supernatants (100 μl) were added to a Maxisorp 96-well plate (ThermoFisher Scientific) coated with anti-CXCL10 capture Ab. Unbind proteins were washed out before adding anti-CXCL10 detection Abs. 1:1000 diluted HRP-conjugated streptavidin (SA-HRP) (Biolegend) was used for the detection of CXCL10. 50 μl of 3, 3′, 5, 5′-tetramethylbenzidine (TMB) (Thermo Fisher Scientific) was added to color development. The reactions were stopped by adding 50 μl H_2_SO_4_. Absorbance was measured using a SpectraMax microplate reader and data were processed by SoftMax pro version 7.1 (Molecular Devices, San Jose, CA, USA).

### Quantitative real time PCR

Total RNAs were extracted from pellet of glioma homogenates with RNeasy plus mini kit (Qiagen, Düsseldorf, Germany) and used for cDNA synthesis with ReverTra Ace^TM^ qPCR RT Master Mix (Toyobo, Osaka, Japan), following the manufacturer’s instruction. Quantitative PCR was conducted with SYBR Green-based quantification (Toyobo) using CFX96 real-time PCR system 3.1 (Bio-Rad, CA, USA). The following primers were used. *Hprt* (F) 5′-GTT GGA TAC AGG CCA GAC TTT GTT G-3′ and (R) 5′-GAG GGT AGG CTG GCC TAT TGG CT-3′; and *Il12b* (F) 5′-GGT GTA ACC AGA AAG GTG CG-3′ and (R) 5′-AAG GTG TCA TGA TGA ACT TAG G-3′. Results were normalized to *Hprt*.

### Histological analysis

Mice were transcardially perfused with 50 ml cold PBS to remove blood from the tissues and 50 ml of cold 4% paraformaldehyde (PFA, pH 7.4, phosphate buffered) to fix tissues. Isolated tissues were fixed in 4% PFA at 4 °C for 16 h and then dehydrated by sinking in 30% sucrose solution for 72 h. Tissues were embedded in OCT solution (Sakura Finetek, Torrance, CA, USA) and sliced to 20 μm thick. Sections were blocked and stained in staining buffer (5% goat serum and 0.3% Triton-X100 (Bio Basic Inc., Amherst, NY, USA) in DPBS) with the following primary antibodies: 1:200 diluted CD31 (2H8), CD3e (SP7) (Thermo), CD169 (3D6.112) (Bio-Rad), NK1.1 (polyclonal) (Bioss, Woburn, MA, USA), and cleaved caspase-3 (Asp175) (Cell Signaling Technology, Danvers, MA, USA). Sections were washed with wash buffer (0.3% Triton-X100 in DPBS) and stained with 1:500 diluted fluorescent-conjugated antibodies; anti-rat IgG-Rhodamine, anti-rabbit IgG-Cy5 and anti-armenian hamster IgG-Alexa Fluor 647 (polyclonal) (Jackson ImmunoResearch, West Grove, PA, USA). For staining of F4/80 or CD45.2, samples were incubated with 1:200 diluted APC-conjugated anti-mouse F4/80 (BM8) antibody (Thermo Fisher Scientific) or APC-conjugated anti-mouse CD45.2 (104) antibody (BD Biosciences) after staining of other antibodies. Samples were mounted with DAPI-containing mounting solution (Abcam). Images were obtained and processed on a LSM800 confocal microscope with ZEN software (Zeiss, Oberkochen, Germany). Images were processed with Fiji software^52^.

### CD169 ligand expression

GL261 cells were irradiated with 40 Gy of γ-ray and incubated for 2 days in DMEM CM to induce apoptosis. To remove CD169-ligand from the cell surface, GL261 cells were incubated in DMEM CM with 0.0008 unit/ml neuraminidase (Roche) for 1 h at 37 °C. Cells were washed and treated with 0.5 μg/ml of CD169-Fc (R&D systems) or mouse IgG2a (Bioxcell) for 30 min at 37 °C. Cells were washed twice with 1% bovine serum containing DPBS and stained with 1:1000 diluted AF647-conjugated rat anti-mouse IgG (Poly4053) (Biolegend) at 4 °C for 30 min. Cells were analyzed by flow cytometry.

### Phagocytosis analysis

For phagocytosis analysis, apoptotic GL261 cells were incubated with 1 μl/ml carboxyfluorescein succinimidyl ester (CFSE) (Thermo Fisher Scientific) in PBS for 20 min at 37 °C. BMDMs were co-cultured with labeled GL261 or red fluorescent-conjugated latex beads (Sigma-Aldrich). To block CD169, BMDMs were incubated with 2 μg/ml of anti-CD169 antibody (3D6.112) (Bio-Rad) or rat IgG2a (RTK2758) (Biolegend) for 30 min at 37 °C before co-culture. Phagocytosis was analyzed by flow cytometry.

### In vitro T cell response

OT-I T cells were isolated from spleens of OT-I mice with the MagniSort™ Mouse CD8 Positive Selection Kit (Thermo Fisher Scientific). BMDMs were treated with anti-CD169 antibody or rat IgG2a for 30 min and incubated with apoptotic GL261-OVA cells (BMDM:GL261 = 1:5) for 6 h. After removal of tumor cells, BMDMs were mixed with T cells at a 1:1 ratio and incubated for 5 days. IFN-γ-producing T cells were analyzed after stimulation with phorbol myristate acetate (50 ng/ml) and ionomycin (1 μg/ml). The concentration of IFN-γ in the supernatant was measured by ELISA, as described^53^. Briefly, culture supernatant was diluted in PBS containing 5% bovine serum and added to Nunc MaxiSorp 96-well plates coated with 1:1000 diluted anti-IFN-γ antibody (XMG1.2) (Biolegend). Unbound proteins were washed off, and IFN-γ was detected with biotinylated 1:1000 diluted anti-IFN-γ antibody (R4-6A2) (Biolegend) and 1:1000 diluted SA-HRP (Biolegend). TMB and H_2_SO_4_ were added sequentially to develop the color and stop the reaction. Absorbance was measured using a SpectraMax microplate reader, and data were processed by SoftMax Pro 7.1 (Molecular Devices).

### Statistical analysis

All statistical analyses except scRNAseq data were performed using GraphPad Prism version 7 (GraphPad Software, Inc., San Diego, CA, USA). Results were expressed as mean ± standard error of the mean (SEM). Differences between groups were analyzed using the unpaired, two-tailed Student’s *t*-test. Survival data were analyzed with the log-rank test. Values of *p* > 0.05 were considered not significant. Adjusted *p* value (p_adj) for multiple comparisons of scRNAseq data were calculated using the FindAllMarkers function. All DEGs from scRNAseq data had p_adj <0.05. The *p* values and p_adj less than 0.0001 were displayed as *p* < 0.0001 or p_adj <0.0001.

### Reporting summary

Further information on research design is available in the Nature Research Reporting Summary linked to this article.

## Supplementary information

Supplementary Information

Reporting Summary

## Data Availability

Single-cell RNA sequencing data have been deposited in NCBI’s Gene Expression Omnibus (GEO). Data for immune cells from WT and CD169-DTR tumor at day 10, CT2A tumor at day 10 and EGFRvIII^+^TP53^−^PTEN^−^ tumor was deposited under accession code GSE201559, and GL261 tumor at day 20 was deposited under accession code GSE200533. Source data are provided with this paper.

## Source data

Source Data
