# Supplementary material for: Blood monocyte-derived CD169+ macrophages contribute to antitumor immunity against glioblastoma
Source: Nat Commun. 2022 Oct 20;13:6211. doi: 10.1038/s41467-022-34001-5 (PMC9585054; doi:10.1038/s41467-022-34001-5)
Supplement: Supplementary file 1 — Supplementary Information [file 41467_2022_34001_MOESM1_ESM.pdf]

Supplementary Information

Title: Blood monocyte-derived CD169^{+} macrophages contribute to antitumor immunity against glioblastoma

Authors: Hyun-Jin Kim[1], Jang Hyun Park[1], Hyeon Cheol Kim[1], Chae Won Kim[1], In Kang[1], Heung Kyu Lee[1,*]

Affiliations: [1]Graduate School of Medical Science and Engineering, Korea Advanced Institute of Science and Technology (KAIST), Daejeon 34141, Republic of Korea

Supplementary Figures

Supplementary Figure legends

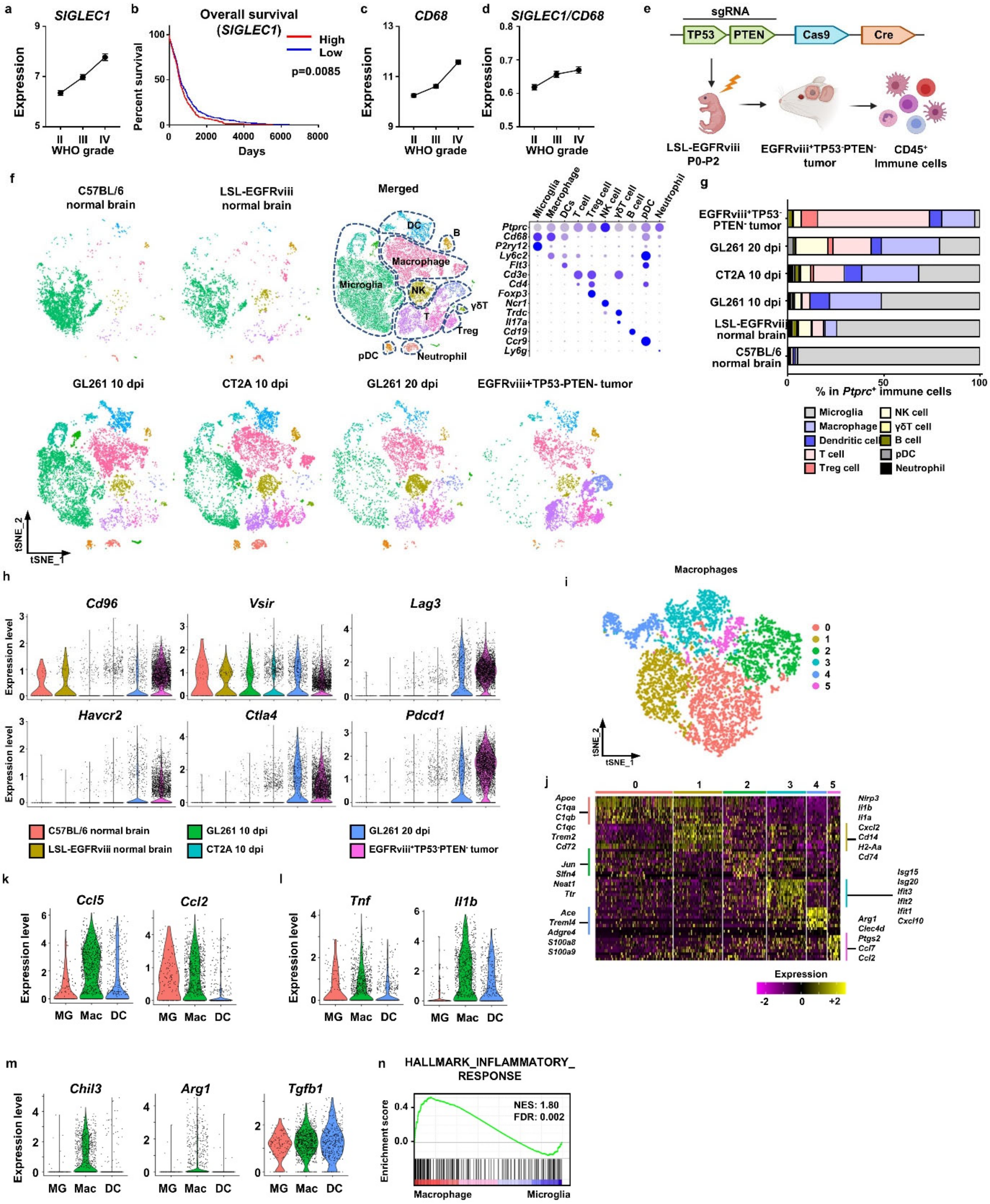

Supplementary Figure 1. CD169 is expressed in inflammatory macrophages in gliomas.

a-d We analyzed human data from The Cancer Genome Atlas Glioblastoma Multiforme and Low Grade Glioma (TCGA-GBMLGG) databases (n = 694). Patients were grouped by their gene expression levels compared to the median. **(a)** *SIGLEC1*, **(c)** *CD68,* and **(d)** *SIGLEC1/CD68* ratio using World Health Organization (WHO) tumor grades. **(b)** Overall survival of patients with high (n = 347) or low (n = 347) according to higher or lower expression than the median (6.82) of *SIGLEC1*. Differences in survival was measured by log-rank

(Mantel-Cox) test.

e Experimental scheme of mouse GBM model induced by genetic modification. Gene transfer of small guide RNA (sgRNA) for tumor protein p53 (*Trp53*) and phosphatase and tensin homolog (*Pten*), and gene coding clustered regularly interspaced short palindromic repeats (CRISPR) associated protein 9 (*Cas9*) and *Cre-recombinase* into the brain ventricle of newborn mice induced epidermal growth factor receptor variant III (*EgfrvIII*) expression, while ablating expression of the tumor suppressor genes, *TP53* and *PTEN*. $CD45^{+}$ immune cells were isolated from engineered tumors that developed in adult mice. Figures were created with BioRender.com.

f-h CD45-expressing immune cells were sorted from tumors from the mouse glioma model or normal mouse brain for single-cell RNA sequencing. **(f)** Immune cells were clustered by their expression of the following marker genes: microglia (*Cd68*$^{+}$*P2ry12*$^{+}$), infiltrated macrophages (*Cd68*$^{+}$*Ly6c2*$^{+}$*Flt3*$^{-}$), dendritic cells (DC; *Flt3*$^{+}$), T cells (*Cd3e*$^{+}$), regulatory T cells (Treg; *Cd3e*$^{+}$*Cd4*$^{+}$*Foxp3*$^{+}$), natural killer cells (NK; *Ncr1*$^{+}$), γδ T cells (*Trdc*$^{+}$*Il17a*$^{+}$), B cells (*Cd19*$^{+}$), plasmacytoid dendritic cells (pDC; *Ccr9*$^{+}$), and neutrophils (*Ly6g*$^{+}$). **(g)** The proportion of immune cells in *Ptprc*$^{+}$ cells from each mouse model. **(h)** Expression levels of inhibitory receptors (*Cd96, Vsir, Lag3, Havcr2, Ctla4,* and *Pdcd1*) in T cell clusters.

i-j Tumor-infiltrating macrophage clusters (GL261, CT2A, and $EGFRvIII^{+}TP53^{-}PTEN^{-}$ tumors) were re-clustered by gene expression.

k-n Gene expression of immune cells from $EGFRvIII^{+}TP53^{-}PTEN^{-}$ tumors. Expression level of **(k)** proinflammatory chemokines (*Ccl5* and *Ccl2*), **(l)** cytokines (*Tnf* and *Il1b*), and **(m)** M2 macrophage markers (*Chil3, Arg1,* and *Tgfb1*) in myeloid cell clusters. **(n)** Gene set enrichment analysis in macrophage and microglia clusters.

Source data are provided as a Source Data file.

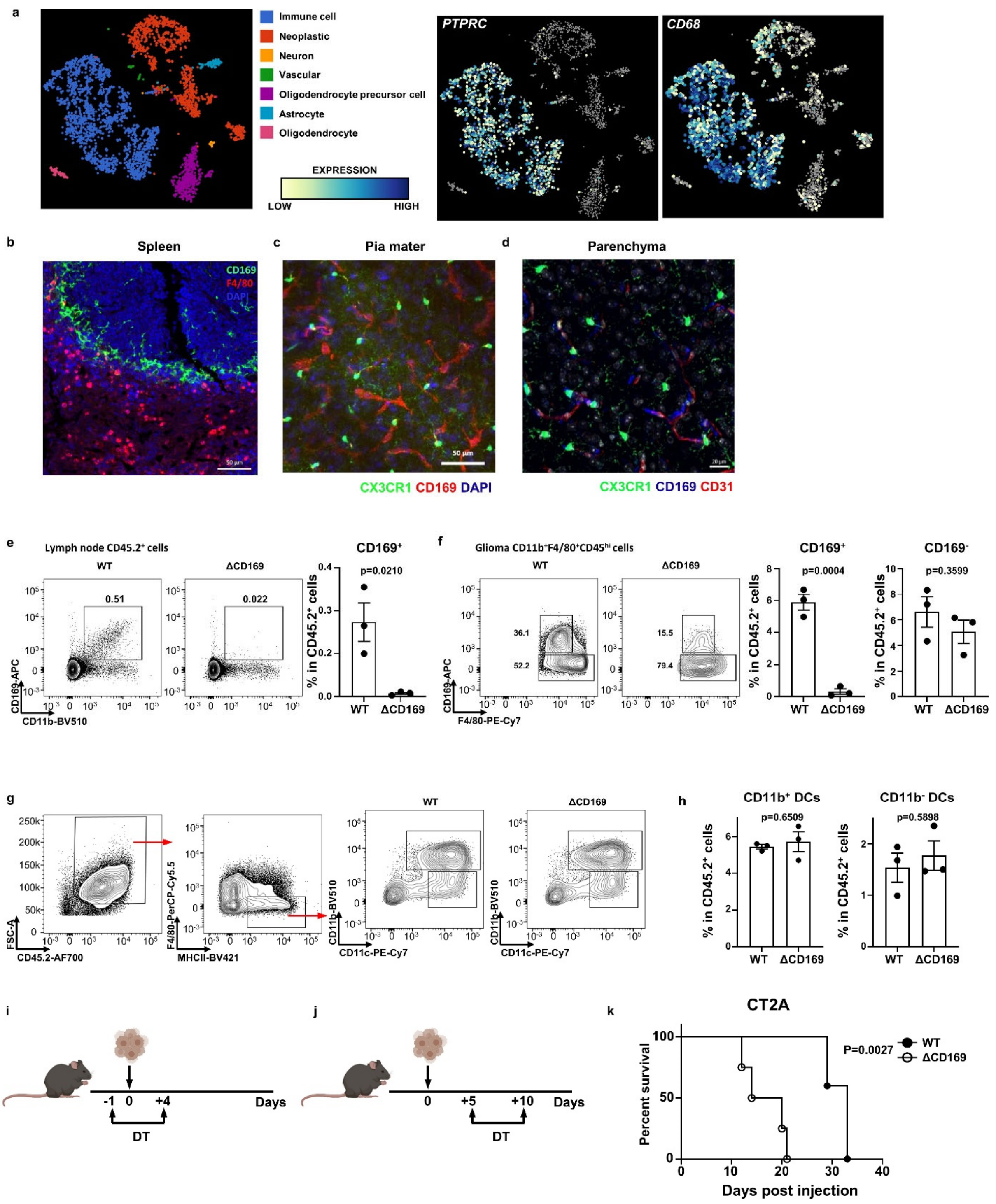

Supplementary Figure 2. CD169^{+} macrophages are in normal tissues but not in brain parenchyma.

a Cell clusters and expression of *PTPRC* and *CD68* in human GBM (GSE84465).

b-d Expression of CD169 in normal **(b)** spleen marginal zone metallophilic macrophages (n = 2), **(c)** brain pia mater (n = 2), and **(d)** perivascular macrophages (n = 2). Data represent two independent experiments. Scale bars indicate **(b-c)** 50 μm and **(d)** 20 μm.

e-h GL261 injected WT (n = 3) and CD169-DTR (n = 3) mice were treated with DT at 20 days aftrer GL261 injection. Macrophages in **(e)** lymph nodes and **(f)** glioma, and DCs **(g-h)** in glioma after DT treatment. Data represent 3 independent experiments. Bars indicate standard error of the mean (SEM). P-values were calculated from an unpaired two-tailed *t*-test.

i-j Experimental scheme for the depletion of cells expressing CD169. **(i)** For depletion of $CD169^+$ cells before tumor formation, 40 ng/g of DT was intraperitoneally injected at -1 and +4 days of GL261 injection. **(j)** For depletion of $CD169^+$ cells after tumor formation, DT was injected at 5 and 10 days after GL261 injection. Figures were created with BioRender.com.

k Survival of mice inoculated with intracranial CT2A cells and intraperitoneal diphtheria toxin (DT; 40 ng per 1 g of mouse body weight). Wild-type (WT, n = 5) and CD169-DTR (n = 4) mice were intraperitoneally injected with DT at −1 and +4 days of CT2A cell injection. Data represent two independent experiments. P-value was calculated with the Log-rank test.

Source data are provided as a Source Data file.

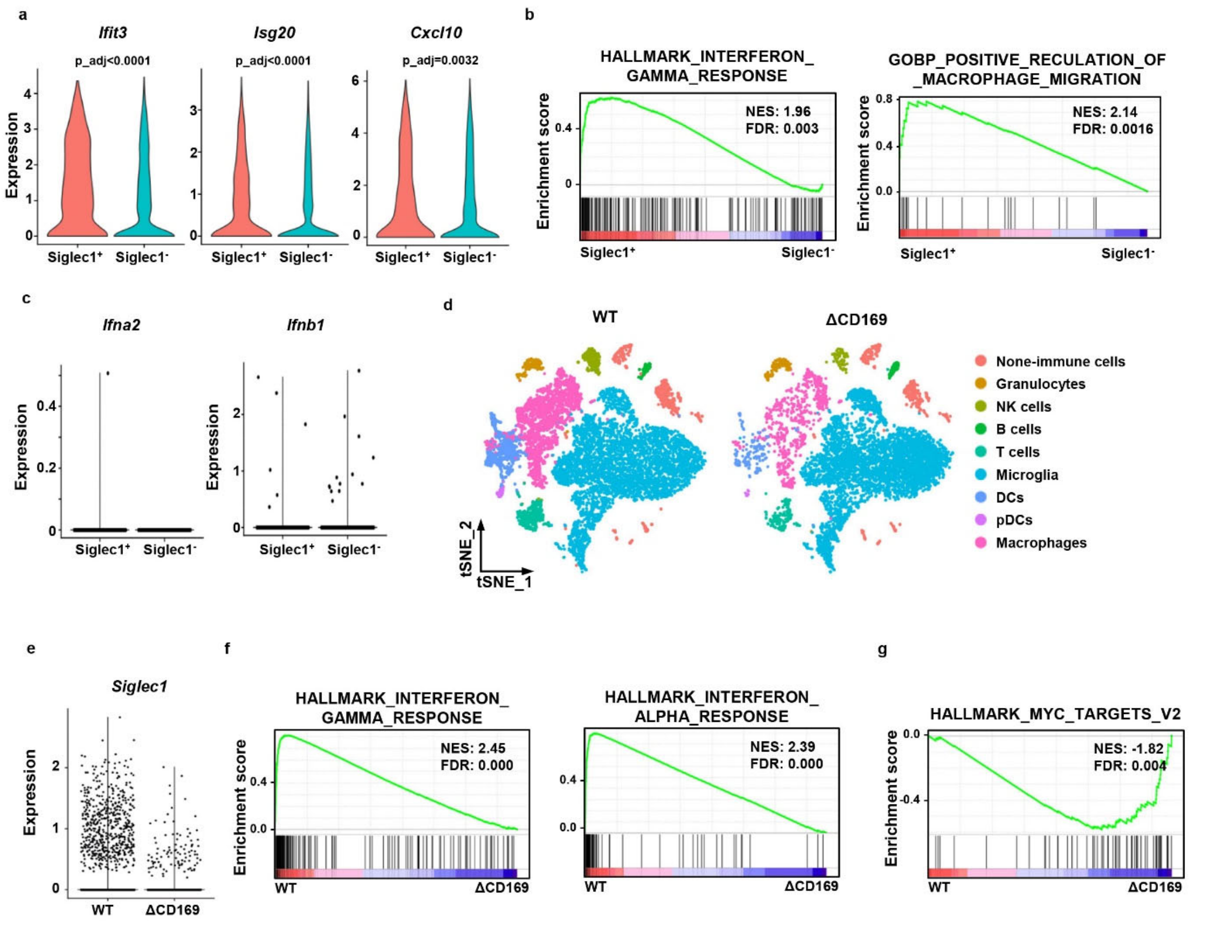

Supplementary Figure 3. CD169 depletion reduces the infiltration and inflammatory gene expression of macrophages.

a-c Macrophages from GL261 tumor samples were clustered by the expression of *Siglec1*. Analysis of **(a)** interferon (IFN)-stimulated gene expression, **(b)** gene set enrichment of IFN-γ response and macrophage migration, and **(c)** type I IFN expression. p_adj indicates adjusted p value.

d CD45.2^{+} immune cells from gliomas of WT and CD169-depleted mice were clustered.

e Expression level of *Siglec1* in the macrophage cluster.

f-g Enriched gene sets in macrophages of **(f)** WT and **(g)** CD169-DTR mice.

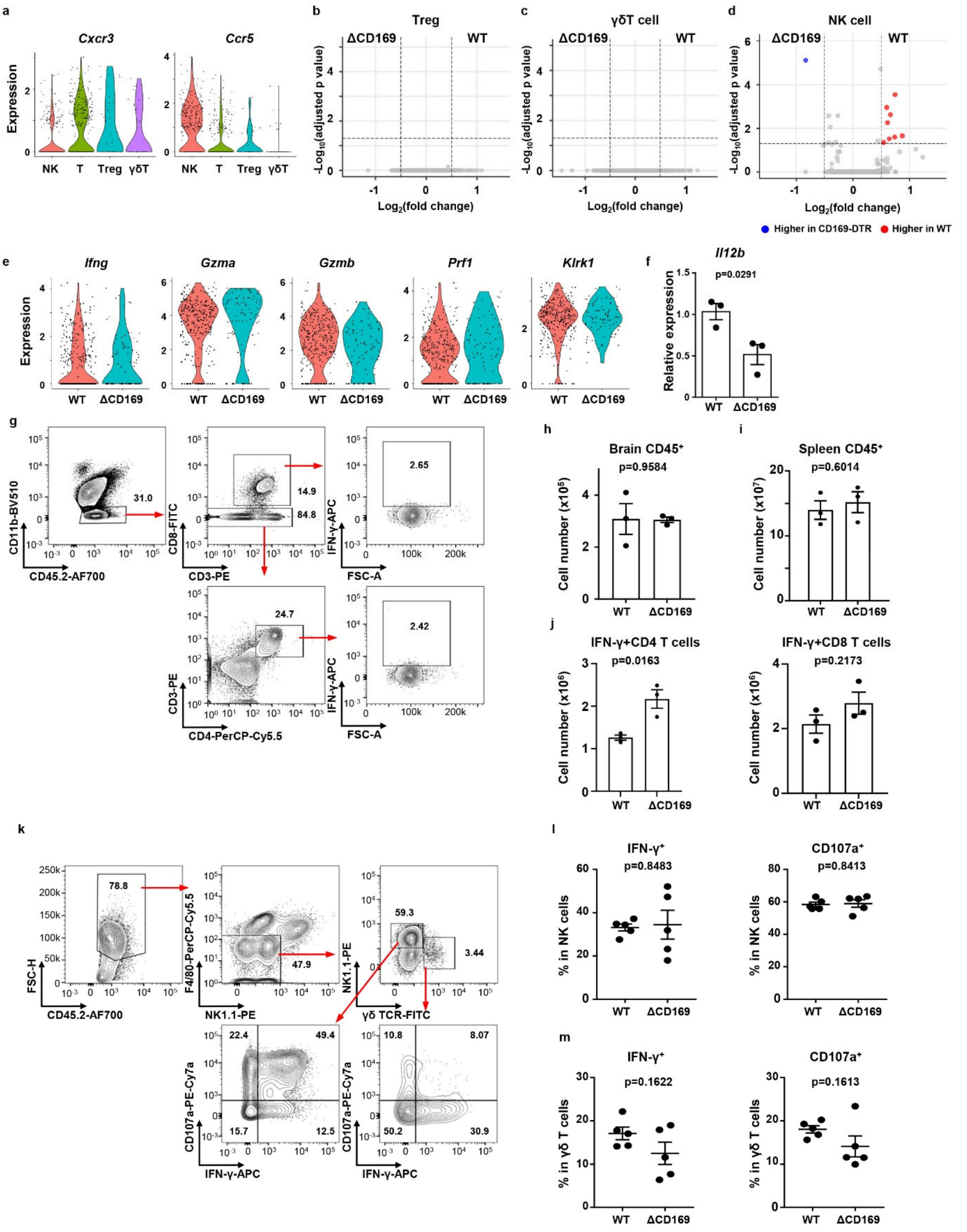

Supplementary Figure 4. CD169 depletion has limited effects on Treg, γδ T cells, NK cells, and splenic T cells.

a Expression of chemokine receptors (*Cxcr3* and *Ccr5*) in the lymphocyte clusters.

b-d DEG analysis of WT and CD169-DTR mice for **(b)** Tregs, **(c)** γδ T cells, and **(d)** NK cells

(Gray dots indicate |Log_2(fold change)| <0.5 or adjusted p-value >0.05).

e Expression of genes associated with activation (*Ifng*, *Gzma*, *Gzmb*, *Prf1,* and *Klrk1*) in the NK cell cluster.

f Expression of *Il12b* mRNA was measured in glioma of biologically independent WT (n = 3) and CD169-DTR (n = 3) mice.

g-j Flow cytometry of immune cells in the brain and spleen of WT (n = 3) and CD169-DTR (n = 3) mice. (**g**) Gating strategy for IFN-γ-expressing CD4 and CD8 T cells in gliomas. The number of $CD45^+$ immune cells in **(h)** brain and **(i)** spleen of mice. **(j)** IFN-γ^+ CD4 and CD8 T cells in the spleen.

k-m Expression of IFN-γ and CD107a in NK cells and γδ T cells in brain tumors of WT (n = 5) and CD169-DTR (n = 5) mice were analyzed by flow cytometry at 10 dpi. **(k)** Gating strategy for NK cells and γδ T cells that expressed IFN-γ and CD107a. IFN-γ^+ or $CD107a^+$ **(l)** NK cells or **(m)** γδ T cells in gliomas were analyzed.

Data are presented as mean values +/- SEM. All p-values were calculated by unpaired two-tailed *t*-test. Data represent two independent experiments.

Source data are provided as a Source Data file.

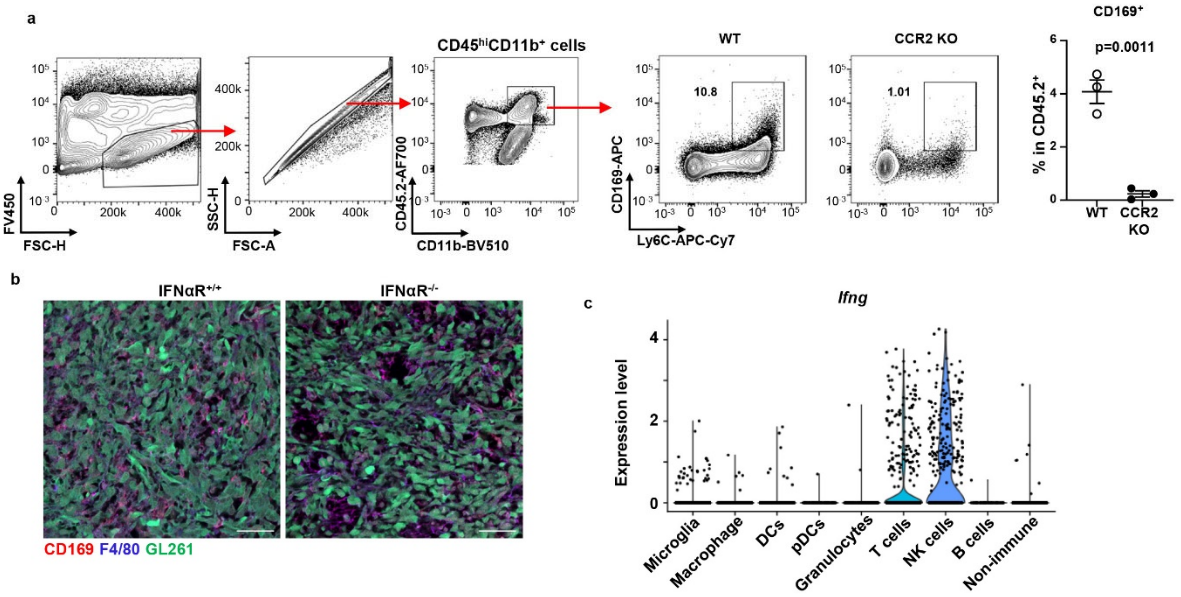

Supplementary Figure 5. Tumor infiltration of CD169$^+$ macrophages is regulated by IFN-γ but not by IFN-α signaling.

a WT (n = 3) and CCR2 knockout (KO) (n = 3) mice were injected with GL261 and analyzed by flow cytometry 15 days after tumor injection. Bold numbers in plots indicate the percentage of gated cells. Bars indicate SEM. P-values were calculated by unpaired two-tailed *t*-test. Data represent two independent experiments.

b WT (n = 3) and IFNαR KO (n = 3) mice were injected with GL261-GFP and analyzed 10 days after tumor injection. Scale bar indicates 50 µm. Data represent two individual experiments.

c Expression of *Ifng* in immune cells isolated 10 dpi of GL261 injection were analyzed.

Source data are provided as a Source Data file.

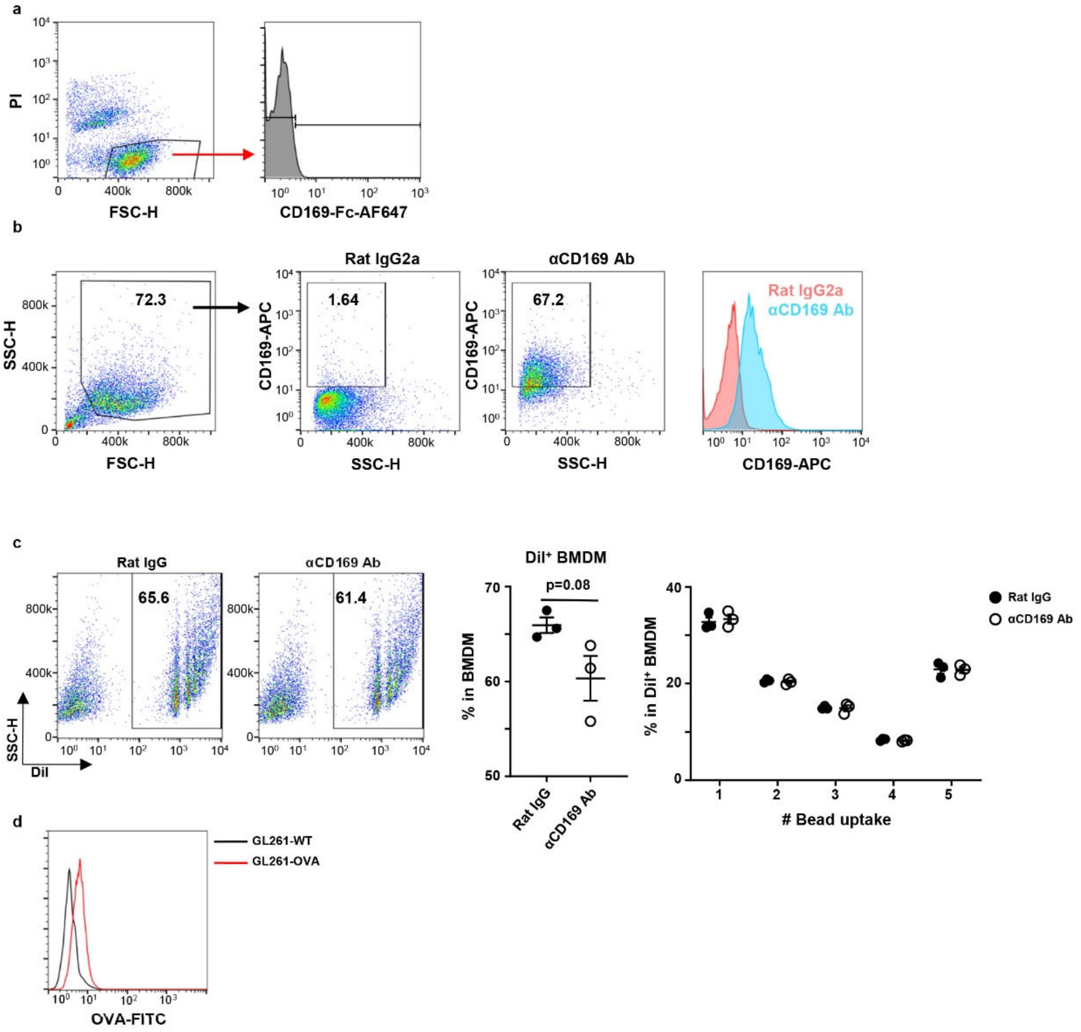

Supplementary Figure 6. Interaction between CD169 on macrophages and CD169 ligand on apoptotic GL261 cells promotes phagocytosis

a Gating strategy for analysis of CD169-ligand on GL261 tumor cells.

b Gating strategy for analysis of CD169 expression on BMDMs.

c BMDMs were treated with anti-CD169 blocking antibody (Ab) or Rat IgG2a isotype control Ab and co-cultured with fluorescently labeled latex bead. Bead uptake by BMDM was analyzed by flow cytometry. Data represent two independent experiments. Bars indicate SEM. P-values were calculated from an unpaired two-tailed *t*-test.

d Expression of OVA in WT GL261 and GL261-OVA cell lines.

Source data are provided as a Source Data file.
