# Supplementary material for: Blood monocyte-derived CD169+ macrophages contribute to antitumor immunity against glioblastoma
Source: Nat Commun. 2022 Oct 20;13:6211. doi: 10.1038/s41467-022-34001-5 (PMC9585054; doi:10.1038/s41467-022-34001-5)
Supplement: Supplementary file 2 — Reporting Summary [file 41467_2022_34001_MOESM2_ESM.pdf]

nature research

Corresponding author(s): Heung Kyu Lee

Last updated by author(s): Sep 14, 2022

Reporting Summary

Nature Research wishes to improve the reproducibility of the work that we publish. This form provides structure for consistency and transparency in reporting. For further information on Nature Research policies, see our Editorial Policies and the Editorial Policy Checklist.

Statistics

For all statistical analyses, confirm that the following items are present in the figure legend, table legend, main text, or Methods section.

n/a	Confirmed	
☐	☒	The exact sample size (*n*) for each experimental group/condition, given as a discrete number and unit of measurement
☐	☒	A statement on whether measurements were taken from distinct samples or whether the same sample was measured repeatedly
☐	☒	The statistical test(s) used AND whether they are one- or two-sided<br>*Only common tests should be described solely by name; describe more complex techniques in the Methods section.*
☒	☐	A description of all covariates tested
☒	☐	A description of any assumptions or corrections, such as tests of normality and adjustment for multiple comparisons
☐	☒	A full description of the statistical parameters including central tendency (e.g. means) or other basic estimates (e.g. regression coefficient) AND variation (e.g. standard deviation) or associated estimates of uncertainty (e.g. confidence intervals)
☐	☒	For null hypothesis testing, the test statistic (e.g. *F*, *t*, *r*) with confidence intervals, effect sizes, degrees of freedom and *P* value noted<br>*Give P values as exact values whenever suitable.*
☒	☐	For Bayesian analysis, information on the choice of priors and Markov chain Monte Carlo settings
☒	☐	For hierarchical and complex designs, identification of the appropriate level for tests and full reporting of outcomes
☐	☒	Estimates of effect sizes (e.g. Cohen's *d*, Pearson's *r*), indicating how they were calculated

Our web collection on statistics for biologists contains articles on many of the points above.

Software and code

Policy information about availability of computer code

Data collection

scRNAseq: Cells were sorted using FACS Aria II (BD Biosciences) with FACSDiva software version 8.0.2 (BD Biosciences). Next-generation sequencing was performed using HiSeqXten (Illumina).

Flow cytometry: Data were collected with FACS Calibur using CellQuest Pro software version 6.0, or with LSRFoltessa X20 using FACSDiva software version 8.0.2.

ELISA data were measured using SpectraMax microplate reader and processed by SoftMac pro version 7.1 (Molecular Devices).

Immunofluorescence: Images were obtained using LSM800 confocal microscopy (Carl Zeiss) with Zen (blue edition) version 2.3 software

Quantitative RT-PCR were performed with CFX Manager™ Software version 3.1 (https://www.bio-rad.com/)

Data analysis

scRNAseq: Cell Ranger pipeline (10X Genomics) was used to generate the expression matrices from single cell RNA sequencing data. Matrices were processed and analyzed with R 3.6.1 software using Seurat v.3 package and EnhancedVolcanoPlot ver.1.13.2 package. GSEA software version 4.0.3. was used for analysis of gene set enrichment using gene set databased MSigDB v7.4. Human GBM scRNAseq data set (GSE84465) was analyzed using BioTuring Bbrowser2 software, version 2.8.22 for Window OS (BioTuring Inf.)

Flow cytometry: Flow Cytometry Standard (FSC) 3.1 files were analyzed with FlowJo version 10 (Treestar)

ELISA: Data were analyzed using SoftMac pro version 7.1 (Molecular Devices).

Immunofluorescence: Zen (blue edition) version 2.3 or 1.1.2 (Carl Zeiss) and Fiji (ImageJ) were used for analysis of confocal microscopy data.

Statistical analysis and visualization of data, except scRNSseq data, were performed with Prism 7.0 (graphPad Software).

Data from quantitative RT-PCR were analyzed with CFX Manager™ Software version 3.1 (https://www.bio-rad.com/)

For manuscripts utilizing custom algorithms or software that are central to the research but not yet described in published literature, software must be made available to editors and reviewers. We strongly encourage code deposition in a community repository (e.g. GitHub). See the Nature Research guidelines for submitting code & software for further information.

Data

Policy information about availability of data

All manuscripts must include a data availability statement. This statement should provide the following information, where applicable:

- Accession codes, unique identifiers, or web links for publicly available datasets
- A list of figures that have associated raw data
- A description of any restrictions on data availability

TCGA lower grade glioma and glioblastoma (TCGA-GBMLGG) data was collected using XENA platform (https://xena.ucsc.edu/).(Figure 1A-F).

Published scRNAseq data of human GBM patients were collected from NCBI GEO (GSE84465) (Figure 2A-B and S2A).

RNA sequencing data of human GBM tissues was collected from Ivy Glioblastoma Atlas Project (http://glioblastoma.alleninstitute.org/) (Figure 2B).

Single cell RNA sequencing data of mouse gliomas were deposited on GEO (GSE201559 and) at the NCBI. (Figure 1c-j, 3a-d, 3f-h, 4a-f, and Supplementary Figure 1f-n, 3a-g, 4a-e and 5c)

Field-specific reporting

Please select the one below that is the best fit for your research. If you are not sure, read the appropriate sections before making your selection.

☒ Life sciences ☐ Behavioural & social sciences ☐ Ecological, evolutionary & environmental sciences

For a reference copy of the document with all sections, see nature.com/documents/nr-reporting-summary-flat.pdf

Life sciences study design

All studies must disclose on these points even when the disclosure is negative.

Sample size	We referred prior experiment of our laboratory to predetermine sample size that proper to identify differences between groups, statistical analyses and reproducibility. The minimal number of animals for each group was 3. Each sample was prepared from biologically independent mouse. All experiments were replicated at least two times. Sample size were referenced previously published article (https://www.nature.com/articles/s41590-020-00860-7)
Data exclusions	No data was excluded.
Replication	All experiments were replicated. The number of replication were described in each figure legend.
Randomization	All mice were grouped by their genotype or treatment. Inside the each grout, mice were randomly assigned. For in vitro experiment, samples were organized into groups according to genotype. Before treatment, samples were randomly allocated to each group.
Blinding	Samples from animals in different groups were collected alternately. Data collection and analysis were blinded from randomized samples.

Reporting for specific materials, systems and methods

We require information from authors about some types of materials, experimental systems and methods used in many studies. Here, indicate whether each material, system or method listed is relevant to your study. If you are not sure if a list item applies to your research, read the appropriate section before selecting a response.

Materials & experimental systems

n/a	Involved in the study	
☐	☒	Antibodies
☐	☒	Eukaryotic cell lines
☒	☐	Palaeontology and archaeology
☐	☒	Animals and other organisms
☒	☐	Human research participants
☒	☐	Clinical data
☒	☐	Dual use research of concern

Methods

n/a	Involved in the study	
☒	☐	ChIP-seq
☐	☒	Flow cytometry
☒	☐	MRI-based neuroimaging

Antibodies

Antibodies used

For flow cytometry
Anti-mouse CD3e-PE-Cy7, (clone 145-2C11)-BD-Cat# 552774
Anti-mouse CD4-PerCP-Cy5.5, (clone RM4.5)-BD-CAT# 561115
Anti-mouse CD8a-FICT, (clone 53-6.7)-Biolegend-Cat# 100705
Anti-mouse CD11b-BV510, (clone M1/70)-BD-Cat# 562950
Anti-mouse CD45.1-PerCP-Cy5.5, (clone A20)-BD-Cat# 560580
Anti-mouse F4/80-PE/Cy7, (clone BM8)-Biolegend-Cat# 123113
Anti-mouse CD45.2-Alexa Fluor 700, (clone 104)-Biolegend-Cat# 109822
Anti-mouse CD192 (CCR2)-PE, (clone SA203G11)-Biolegend-Cat# 150609
Anti-mouse Ly6C-APC/Cy7, (clone HK1.4)-Biolegend-Cat# 128026
Anti-mouse CD169 (Siglec1)-APC, (clone 3D6.112)-Biolegend-Cat# 142418
Anti-mouse CD107a (LAMP1)-PE-Cy7, (clone 1D4B)-BD-Cat# 560647
Anti-mouse IFN-γ-APC, (clone XMG1.2)-Biolegend-Cat# 505810
Anti-mouse IFN-γ-PE, (clone XMG1.2)-Biolegend-Cat# 505807
Anti-mouse NK1.1-PE, (clone PK136)-Biolegend-Cat# 108708
Anti-mouse F4/80-PerCP-Cy5.5, (clone BM8)-ThermoFisher Scientific-Cat# 45-4801-82
Anti-mouse γδ TCR-FITC, (clone GL3)-ThermoFisher Scientific-Cat# 11-5711-82
PE/Cy7 Rat IgG2a, κ isotype control, (clone RTK2758)-Biolegend-Cat# 400521
PE Rat IgG1, κ isotype control, (clone RTK2071)-Biolegend-Cat# 400408
APC Rat IgG1, κ isotype control, (clone RTK2071)-Biolegend-Cat# 400412
Anti-mouse CD45.2-APC, (clone 104)-BD-Cat# 558702
Anti-mouse IgG-Alexa Fluor 647, (clone Poly4053)-Biolegend-Cat# 405322
Anti-Ovalbumin-FITC, (polyclonal)-Abcam-Cat# 85584
Anti-mouse MHCII(I-A/I-E)-BV421, (clone M5/114)-BD-Cat# 562564
Anti-mouse CD11c-PE-Cy7, (clone N418)-Biolegend-Cat# 117318

Immunofluorescence
Anti-mouse CD31, (clone 2H8)-Thermo Scientific-Cat# MA3105
Anti-mouse CD169, (clone 3D6.112)-Bio-Rad-Cat# MCA884GA
Anti-mouse CD3, (clone SP7)-Thermo Scientific-Cat# MA5-14524
Anti-mouse F4/80-APC (clone BM8)-eBioscience-Cat# 17-4801-82
Anti-mouse CD45.2-APC, (clone 104)-BD-Cat# 558702
Anti-mouse NK1.1 (polyclonal)-Bioss-Cat# BS-4682R
Anti-mouse cleaved caspase-3 (clone Asp175)-Cell Signaling Technology-Cat# 9661
Anti-rat IgG-Rhodamine (TRITC), (ployclonal)-Jackson ImmunoResearch -Cat# 112-025-003
Anti-rabbit IgG-Cy5, (Polyclonal)-Jackson ImmunoResearch-Cat# 111-175-144
Anti-Armenian hamster IgG-Alexa Fluor 647, (polyclonal)-Jackson ImmunoResearch-Cat# 127-605-160

in vivo/in vitro depletion/blockade
mouse IgG2a, isotype control, (clone C1.18.4)-BioXcell-Cat# BE0085
anti-mouse NK1.1, (clone PK136)-BioXcell-Cat# BE0036
Anti-mouse CD169, (clone 3D6.112)-Bio-Rad-Cat# MCA884GA
Rat IgG2a, κ Isotype Ctrl Antibody (RTK2758)-Biolegend-Cat# 400501

Validation

Antibodies are commercially available and have been validated by manufacturers except anti-CD16/32 (clone 2.4G2).
Anti-CD16/32 (2.4G2) was purified from supernatant of hybridoma 2.4G2(ATCC, HB-197) and validated by flow cytometry by staining other 2.4G2 antibody. Titer was determined by serial dilution.
Specific validation information for commerciall antibodies is described in website:

Flow cytometry
Anti-mouse CD3e-PE-Cy7, (clone 145-2C11) https://www.bdbiosciences.com/eu/applications/research/t-cell-immunology/th-1-cells/surface-markers/mouse/pe-cy7-hamster-anti-mouse-cd3e-145-2c11/p/552774
Anti-mouse CD4-PerCP-Cy5.5, (clone RM4.5) https://www.bdbiosciences.com/en-ca/products/reagents/flow-cytometry-reagents/research-reagents/single-color-antibodies-ruo/percp-cy-5-5-rat-anti-mouse-cd4.561115
Anti-mouse CD8a-FICT, (clone 53-6.7)-Biolegend-Cat# 100705 https://www.biolegend.com/fr-ch/search-results/fitc-anti-mouse-cd8a-antibody-153
Anti-mouse CD11b-BV510, (clone M1/70) https://www.bdbiosciences.com/eu/applications/research/stem-cell-research/mesenchymal-stem-cell-markers-bone-marrow/mouse/negative-markers/bv510-rat-anti-cd11b-m170/p/562950
Anti-mouse CD45.1-PerCP-Cy5.5, (clone A20) https://www.bdbiosciences.com/eu/applications/research/stem-cell-research/cancer-research/mouse/percp-cy55-mouse-anti-mouse-cd451-a20/p/560580
Anti-mouse F4/80-PE/Cy7, (clone BM8) https://www.biolegend.com/en-us/products/pe-cyanine7-anti-mouse-f4-80-antibody-4070
Anti-mouse CD45.2-Alexa Fluor 700, (clone 104) https://www.biolegend.com/en-us/products/alexa-fluor-700-anti-mouse-cd45-2-antibody-3393
Anti-mouse CD192 (CCR2)-PE, (clone SA203G11) https://www.biolegend.com/en-us/products/pe-anti-mouse-cd192-ccr2-antibody-13336
Anti-mouse Ly6C-APC/Cy7, (clone HK1.4) https://www.biolegend.com/en-us/products/apc-cyanine7-anti-mouse-ly-6c-antibody-6758
Anti-mouse CD169 (Siglec1)-APC, (clone 3D6.112) https://www.biolegend.com/en-us/products/apc-anti-mouse-cd169-siglec-1-antibody-11851
Anti-mouse CD107a (LAMP1)-PE-Cy7, (clone 1D4B) https://www.biolegend.com/en-us/products/apc-anti-mouse-cd107a-lamp-1-antibody-6081
Anti-mouse IFN-γ-APC, (clone XMG1.2) https://www.biolegend.com/en-us/products/apc-anti-mouse-ifn-gamma-antibody-993

Anti-mouse IFN-γ-PE, (clone XMG1.2) https://www.biolegend.com/nl-nl/products/pe-anti-mouse-ifn-gamma-antibody-997
Anti-mouse NK1.1-PE, (clone PK136) https://www.biolegend.com/en-us/products/pe-anti-mouse-nk-1-1-antibody-431
Anti-mouse F4/80-PerCP-Cy5.5, (clone BM8) https://www.thermofisher.com/antibody/product/F4-80-Antibody-clone-BM8-Monoclonal/45-4801-82
Anti-mouse γδ TCR-FITC, (clone GL3) https://www.thermofisher.com/antibody/product/TCR-gamma-delta-Antibody-clone-eBioGL3-GL-3-GL3-Monoclonal/11-5711-82
PE/Cy7 Rat IgG2a, κ isotype control, (clone RTK2758)-https://www.biolegend.com/en-gb/products/pe-cyanine7-rat-igg2a-kappa-isotype-ctrl-1935
APC Rat IgG1, κ isotype control, (clone RTK2071) https://production.biolegend.com/en-us/search-results/apc-rat-igg1-kappa-isotype-ctrl-1826?GroupID=GROUP29
PE Rat IgG1, κ isotype control, (clone RTK2071) https://production.biolegend.com/en-us/products/pe-rat-igg1-kappa-isotype-ctrl-1830
Anti-mouse CD45.2-APC, (clone 104) https://www.bdbiosciences.com/us/applications/research/stem-cell-research/cancer-research/mouse/apc-mouse-anti-mouse-cd452-104/p/558702
Anti-mouse IgG-Alexa Fluor 647, (clone Poly4053) https://production.biolegend.com/en-us/products/alexa-fluor-647-goat-anti-mouse-igg-minimal-x-reactivity-9283
Anti-Ovalbumin-FITC, (polyclonal) https://www.abcam.com/ovalbumin-antibody-fitc-ab85584.html
Anti-mouse MHCII(I-A/I-E)-BV421, (clone M5/114) https://www.bdbiosciences.com/en-us/products/reagents/flow-cytometry-reagents/research-reagents/single-color-antibodies-ruo/bv421-rat-anti-mouse-i-a-i-e.562564
Anti-mouse CD11c-PE-Cy7, (clone N418) https://www.biolegend.com/en-ie/products/pe-cyanine7-anti-mouse-cd11c-antibody-3086

Immunofluorescence
Anti-mouse CD31, (clone 2H8) https://www.thermofisher.com/antibody/product/CD31-Antibody-clone-2H8-Monoclonal/MA3105
Anti-mouse CD169, (clone 3D6.112) https://www.bio-rad-antibodies.com/monoclonal/mouse-cd169-antibody-3d6-112-mca884.html?f=purified
Anti-mouse CD3, (clone SP7) https://www.thermofisher.com/antibody/product/CD3e-Antibody-clone-SP7-Monoclonal/MA5-14524
Anti-mouse F4/80-APC (clone BM8) https://www.thermofisher.com/antibody/product/F4-80-Antibody-clone-BM8-Monoclonal/17-4801-82
Anti-mouse CD45.2-APC, (clone 104)-BD-Cat# 558702 https://www.bdbiosciences.com/ko-kr/products/reagents/flow-cytometry-reagents/research-reagents/single-color-antibodies-ruo/apc-mouse-anti-mouse-cd45-2.558702
Anti-mouse NK1.1, (polyclonal)-Bioss-Cat# BS-4682R https://www.biossusa.com/products/bs-4682r
Anti-mouse cleaved caspase-3, (clone Asp175)-Cell Signaling Technology-Cat# 9661 https://www.cellsignal.com/products/primary-antibodies/cleaved-caspase-3-asp175-antibody/9661
Anti-rat IgG-Rhodamine (TRITC), (ployclonal) https://www.jacksonimmuno.com/catalog/products/112-025-003
Anti-rabbit IgG-Cy5, (Polyclonal) https://www.jacksonimmuno.com/catalog/products/111-175-144
Anti-Armenian hamster IgG-Alexa Fluor 647, (polyclonal) https://www.jacksonimmuno.com/catalog/products/127-605-160

in vivo/in vitro depletion/blockade
InVivoMAb mouse IgG2a, isotype control, (clone C1.18.4) https://bxcell.com/product/invivomab-mouse-igg2a-isotype-control-unknown-specificity/
InVivoMAb anti-mouse NK1.1, (clone PK136) https://bxcell.com/product/nk-1-1/
Anti-mouse CD169, (clone 3D6.112) https://www.bio-rad-antibodies.com/static/datasheets/mca88/mouse-cd169-antibody-3d6-112-mca884ga.pdf
Rat IgG2a, κ Isotype Ctrl Antibody (RTK2758) https://www.biolegend.com/en-us/products/purified-rat-igg2a-kappa-isotype-ctrl-1845?GroupID=GROUP29

Eukaryotic cell lines

Policy information about cell lines

Cell line source(s)	The GL261 mouse glioma cell line and GFP-expressing GL261 (GL261-GFP) were provided by Dr. Injune Kim (KAIST). GL261 cell line was origined from K. Plate (Goethe University Medical School, Frankfurt, Germany). GL261-GFP was made in laboratory of Dr. Injune Kim. We purchased the CT2A mouse glioma cell line (Sigma-Aldrich, St. Louis, MO, USA) and the Lenti-X™ 293T cell line (Takara Bio, Kusatsu, Japan). GL261-OVA cell line was constructed in the laboratory using lentiviral transduction of OVA gene. L929 cell line producing M-CSF was provided by A. Iwasaki (Yale University, New Haven, CT, USA).
Authentication	Authentication of GL261 cells, CT2A, L929 and 293T cell line was not performed. GFP expression of GL261-GFP and OVA expression of GL261-OVA were confirmed by flow cytometry. M-CSF production from L292 cells were confirmed through differentiation of bone marrow derived macrophages. cell lines with low passage number were utilized.
Mycoplasma contamination	No mycoplasma contamination was confirmed with e-Myco plus Mycoplasma PCR kit (Intron Biotechnology)
Commonly misidentified lines (See ICLAC register)	None.

Animals and other organisms

Policy information about studies involving animals; ARRIVE guidelines recommended for reporting animal research

Laboratory animals	8-week-old C57BL/6 mice (Female and male) were purchased from KAIST Laboratory Animal Resource Center and DBL Co. Ltd (Korea). CD169-DTR (Siglec1tm1(HBEGF)Mtka) mice were originally generated and kindly provided by Dr. Masato Tanaka (Tokyo University of Pharmacy and Life Sciences, Japan). IFN-γ-/- (B6.129S7-Ifngtm1Ts/J, Stock No: 002287), IFN-αR-/-(B6(Cg)-Ifnar1tm1.2Ees/J, Stock No: 028288), CX3CR-1GFP (B6.129P2(Cg)-Cx3cr1tm1Litt/J, Stock No: 005582), CD45.1 (B6.SJL-Ptprca Pepcb/BoyJ, Stock No: 002014), CCR2 KO (B6.129S4-Ccr2tm1Ifc/J, Stock No: 004999) and OT-I (C57BL/6-Tg [TcraTcrb]1100Mjb/J, Stock No:

	003831) mice were purchased from The Jackson Laboratory. LSL-EGFRvIII mice (FVB strain background) were provided by Dr. Jeong Ho Lee (KAIST, Korea) and backcrossed to C57BL/6 mice. Mice were housed in a specific pathogen-free (SPF) facility at KAIST, in a 12h/12h light/dark cycle at 18-24 degrees Celsius and 30-70% of humidity range. 8-week-old female mice were used for parabiosis experiment. 16-week-old female mice were used for single cell RNA sequencing of EGFRviii+TP53-PTEN- tumor. 8 or 12 week old Male mice were used for other experiments.
Wild animals	None.
Field-collected samples	None.
Ethics oversight	All procedures in this study were performed in accordance with guidelines and protocols (KA2017-41, KA2019-65 and KA2021-048) approved by the Institutional Animal Care and Use Committee (IACUC) of KAIST.

Note that full information on the approval of the study protocol must also be provided in the manuscript.

Flow Cytometry

Plots

Confirm that:

☒ The axis labels state the marker and fluorochrome used (e.g. CD4-FITC).

☒ The axis scales are clearly visible. Include numbers along axes only for bottom left plot of group (a 'group' is an analysis of identical markers).

☒ All plots are contour plots with outliers or pseudocolor plots.

☒ A numerical value for number of cells or percentage (with statistics) is provided.

Methodology

Sample preparation	Tissues were minced and digested with with 2 mg/ml collagenase IV and 30 μg/ml DNase I at 37℃ for 30 min, and passed through a 70 μm strainer. To remove non-immune cells from tumor or brain samples, cells were resuspended in 4 ml of 30% Percoll and loaded onto 3 ml of 70% Percoll. Immune cells and myelin were separated by centrifugation. Cells were treated with ACK lysis buffer for 5 min at RT to remove residual red blood cells. Single cells were resuspended in staining buffer (DPBS with 1% bovine serum and 1% penicillin-streptomycin) with anti-CD16/32 (clone 2.4G2, ATCC HB-197) to block Fc receptors and stained with antibody mixture for 30 min in dark on ice.
Instrument	Cells were aquired with LSR Fortessa X-20 or FACS Calibur (BD Biosciences)
Software	BD FACSDiva or Cellquest pro (BD Biosciences) was used to acquire data. Data were analyzed with Flowjo v10 (Treestar)
Cell population abundance	100,000 of PI-negative and CD45.2-positive cells from tumors or normal brain were purified. Purity was >90%.
Gating strategy	Propidium iodide or Fixable Viability Stain 450 negative cells were gated as live cells. Detailed cell population were determined by markers listed below.<br>Macrophage: CD45-hi/CD11b-pos/F4/80-pos/CCR2-pos<br>Microglia: CD45-low/CD11b-pos/F4/80-pos/CCR2-neg<br>CD4 T cell: CD45-pos/CD3e-pos/CD4-pos<br>CD8 T cell: CD45-pos/CD3e-pos/CD8-pos<br>NK cell: CD45-pos/F4/80-neg/NK1.1-pos<br>gd T cell: CD45-pos/F4/80-neg/gdTCR-pos

☒ Tick this box to confirm that a figure exemplifying the gating strategy is provided in the Supplementary Information.
